# Multi-Platform Metabolomic and Sensory Profiling Reveals Cultivar-Dependent Volatile, Taste, and Antioxidant Chemotypes in Guava Leaf Tea

**DOI:** 10.3390/foods15203616

**Published:** 2026-10-11

**Authors:** Songqiong Ma, Shimiao Chen, Lin Ning, Jingsheng Fan, Jingwen Ai, Haojun Chen, Jiang He, Qinyu Lu, Gan-Lin Chen

**Affiliations:** 1 Guangxi Subtropical Crops Research Institute, Guangxi Academy of Agricultural Sciences, Nanning 530001, China; 2 Key Laboratory of Quality and Safety Control for Subtropical Fruit and Vegetable, Ministry of Agriculture and Rural Affairs, Nanning 530001, China; 3 Guangxi Key Laboratory of Quality and Safety Control for Subtropical Fruits, Guangxi Subtropical Crops Research Institute, Nanning 530001, China

**Keywords:** guava leaf tea, cultivar differentiation, volatile metabolomics, electronic tongue, non-targeted LC–MS, antioxidant capacity

## Abstract

Guava (*Psidium guajava* L.) leaves are increasingly processed into caffeine-free herbal teas, valued for their rich phenolic composition and cultivar-dependent sensory profile. However, no systematic study has yet resolved how cultivar identity shapes the volatile, non-volatile, and functional dimensions of the finished tea. Here, eight guava tea cultivars were profiled using a multi-platform approach combining electronic nose sensing, headspace GC–MS, electronic-tongue analysis, non-targeted and targeted LC–MS metabolomics, and physicochemical indices. The eight cultivars were described by three volatile composition patterns: (i) a monoterpene-rich group, most pronounced in ‘Yanzhi Hong’, (ii) a compositionally diverse non-monoterpene group dominated by sesquiterpene esters and alkanes, and (iii) a group with a lower abundance of most discriminant volatiles—a pattern consistent across electronic nose sensor responses and GC–MS compound-level data. Electronic-tongue profiling identified largely independent instrumental taste-response modules. Instrumental astringency showed a robust, cross-platform-supported association with cultivars enriched in flavonoid glycosides (sparse partial least squares [sPLS] Q^2^ = 0.733–0.900), whereas bitterness was statistically detectable but compositionally unresolved. Antioxidant (DPPH) capacity was only weakly associated with total phenolic and flavonoid contents (r = 0.188 for each), indicating that bulk phenolic indices alone did not adequately explain cultivar-level DPPH variation. Integrating these results into a candidate pathway-output framework indicated that aroma, taste, and antioxidant-related quality are shaped by partially independent metabolic axes rather than a single quality gradient. These findings provide a candidate-based, cultivar-resolved framework for guava leaf tea quality and identify candidate cultivars suited to terpene-rich, non-monoterpene, or phenolic-retention-oriented product development.

## 1. Introduction

Guava (*Psidium guajava* L., Myrtaceae) is an evergreen shrub or small tree widely cultivated throughout tropical and subtropical regions, where both its fruit and leaves have long been valued as food and folk-medicine resources [1]. In recent years, guava leaves have attracted growing interest as a raw material for caffeine-free herbal infusions, motivated by a favorable phenolic and flavonoid profile that distinguishes them from orthodox Camellia sinensis teas while still delivering a distinctive, cultivar-dependent sensory experience [2,3]. The characteristic aroma of guava leaf is carried by a structurally diverse volatile fraction in which β-caryophyllene, limonene, and (E)-nerolidol are frequently reported among the major constituents, together with smaller contributions from other sesquiterpene alcohols, sulfur-containing volatiles, and lipid- or carotenoid-derived compounds [4,5,6]. On the non-volatile side, hydrolyzable ellagitannins such as sanguin H-11 and casuarinin, characteristic flavonol glycosides such as guajavarin and avicularin, and other quercetin derivatives have been associated with the astringent taste and the antioxidant, anti-hyperglycemic, and anti-inflammatory activities most frequently attributed to guava leaf products [7,8]. When leaves are converted into a beverage, drying and other thermal steps inevitably alter part of the volatile fraction and may modify phenolic constituents through enzymatic oxidation and heat-driven transformations; however, a cultivar-informative share of both compartments may remain detectable after processing and infusion preparation and has been shown to differ across guava germplasm [1,2]. Tea quality therefore reflects the coexistence of two chemically independent compartments—a non-volatile domain governing taste- and function-related attributes and a volatile domain governing aroma—both of which are increasingly recognized as cultivar-dependent rather than uniform across *P. guajava* germplasm [9,10].

Existing cultivar comparisons have documented substantial variation in total phenolic content (TPC), total flavonoid content (TFC), and antioxidant capacity [11,12,13], yet they have relied on bulk indices that cannot resolve how individual volatile and non-volatile metabolites jointly shape the aroma, taste, and functional differences perceived in the brewed tea.

Tea quality is inherently multidimensional, integrating aroma, taste, and functional attributes that are mediated by largely distinct chemical classes and require different analytical approaches [14,15]. Aroma depends on a complex mixture of volatiles whose perceived intensity reflects concentration, odor threshold, and interactions among constituents, not the abundance of any single compound [16,17]. Taste similarly spans multiple axes—astringency, bitterness, umami, sweetness—each driven by different non-volatile metabolites [12,18,19]; in guava leaf, astringency has been linked to ellagitannins and flavonol glycosides such as guajavarin and avicularin [20], while umami and sweetness track only loosely with amino acid and soluble sugar contents [21,22]. Antioxidant capacity, in turn, is imperfectly predicted by bulk phenolic or flavonoid indices, because DPPH-type assays integrate contributions from numerous compounds whose structures and relative abundances jointly determine net activity [2,8]. Resolving cultivar-dependent quality differences therefore requires an analytical framework capable of capturing aroma, taste, and antioxidant-related traits simultaneously rather than in isolation.

Research on the chemical basis of guava leaf quality has accumulated over the past two decades but remains uneven in scope and integration. Early studies established the foundational chemical inventory of the leaf, identifying phenolic acids, flavonol glycosides, triterpenoid acids, and essential-oil constituents and documenting their antioxidant, anti-hyperglycemic, and anti-inflammatory activities in vitro [1,2,8]. Subsequent cultivar surveys extended this work by comparing accessions or varieties on the basis of bulk indices in fresh leaves—total phenolic content, total flavonoid content, and DPPH or ABTS scavenging capacity—and consistently found that cultivar identity influences these parameters [11,13,23]. A limited number of studies have begun to examine processing effects: Chang et al. [24] developed a manufacturing protocol for guava leaf tea—comprising early-dawn plucking, withering, rolling, optional fermentation, and low-temperature drying—and reported that the resulting product retained a distinctive polyphenolic profile, including quercetin glycosides, ellagic acid, and catechins. However, that study examined a single cultivar and a single chemical domain; no study has yet compared multiple guava cultivars after processing into tea, nor assessed how cultivar identity interacts with a common processing protocol to shape the finished product’s volatile, non-volatile, and sensory characteristics. This stands in contrast to Camellia sinensis tea, where the interplay between cultivar and processing has been explicitly demonstrated: He et al. [25] showed that both cultivar identity and processing mode significantly shape the volatile aroma system of oolong tea, and metabolomics-driven cultivar comparisons have become an established strategy for linking compound-level variation to quality attributes [26,27]. For guava leaf tea, no comparable cultivar-resolved, multi-platform investigation has been reported. As a result, it remains unclear which volatile metabolites define the aroma chemotypes that distinguish one cultivar from another after processing, which non-volatile compounds underlie taste differences and whether conventional amino acid or soluble sugar proxies are adequate to account for them, and whether the antioxidant capacity of the brewed tea reflects the bulk accumulation of phenolics and flavonoids or the contribution of specific metabolite subsets that bulk-index comparisons simply cannot resolve. Furthermore, no attempt has yet been made to integrate these independent lines of evidence into a candidate pathway–output framework linking metabolic variation to the sensory and functional phenotype of the brewed tea. Without addressing these gaps, cultivar selection and quality improvement for guava leaf tea remain largely empirical.

To address these gaps, we combined electronic nose and HS-GC–MS profiling for aroma, electronic tongue and UHPLC–QTOF-MS for taste-related variation, targeted HPLC for key phenolic and flavonoid compounds, and physicochemical assays for antioxidant- and processing-related traits [2,11,28,29,30]. This design enables a cross-platform comparison of cultivar-associated chemical, sensory, and functional patterns without implying direct mechanistic causation.

In this study, we compared guava leaf teas prepared from eight cultivars—‘Xigua’, ‘Xigua No. 2′, ‘Yanzhi Hong’, ‘Jindou Xiang’, ‘Xinshiji’, ‘Hongbaoshi’, ‘Wuzi’, and ‘Fenhongmi’—using a multi-platform analytical framework comprising electronic nose, headspace GC–MS, electronic tongue, untargeted UHPLC–QTOF-MS metabolomics, targeted HPLC quantification of key flavonoids and phenolic compounds, and a panel of antioxidant- and processing-related physicochemical assays (DPPH scavenging capacity, TPC, TFC, vitamin C, and polyphenol oxidase activity). We aimed to (i) characterize cultivar-dependent volatile aroma profiles and identify the chemotypes that distinguish cultivar groups; (ii) resolve non-volatile metabolite groupings and link them to electronic-tongue taste variation, with particular attention on astringency, umami, and bitterness; (iii) determine whether antioxidant capacity is governed by bulk phenolic accumulation or by specific candidate metabolites; and (iv) integrate the evidence across platforms to construct a candidate pathway–output framework linking metabolic variation to the sensory and functional phenotype of the brewed tea. By assembling cultivar-resolved, compound-level evidence across the volatile, non-volatile, sensory, and functional dimensions within a single study, this work seeks to move guava leaf tea quality evaluation beyond bulk-index comparison toward a candidate-based understanding of how cultivar identity shapes beverage quality.

## 2. Materials and Methods

### 2.1. Plant Materials and Tea Preparation

Eight guava (*Psidium guajava* L.) cultivars—Fenhongmi (FHM), Xigua (XG), Yanzhi Hong (YZH), Jindou Xiang (JDX), Hongbaoshi (HBS), Wuzi (WZ), Xinshiji (XSJ), and Xigua No. 2 (XG2)—were collected from the Guava Germplasm Repository of the Ministry of Agriculture and Rural Affairs (Nanning, Guangxi, China). Three independent biological replicates (500 g of fresh leaves each) were sampled per cultivar from healthy, pest-free, uniformly developing plants of the same physiological age (18 months old). Nine trees were randomly selected per cultivar from the germplasm repository and pooled into three replicates (three trees per replicate) to reduce inter-individual variation. To standardize leaf maturity, only the first two fully expanded leaves immediately below the apical bud were harvested, representing young, fully expanded leaves at a uniform early developmental stage. Sampling was conducted in August 2024 on a single day. Samples were transported to the laboratory immediately after harvest, cleaned of debris, rinsed with deionized water, and air-dried until the leaf surface was free of moisture.

Fresh leaves were processed into guava leaf tea following a standard green tea procedure: withering (25 ± 1 °C, 60 ± 5% relative humidity, 4 h, until soft and pliable), fixing (120 °C, 3 min, until dark green with no grassy odor), rolling (25 rpm, 15 min, until tightly rolled with slight tea juice exudation), and drying (80 °C, to ≤6% moisture). Finished teas were sealed in aluminum-laminate bags and stored at 4 °C in the dark for seven days prior to analysis. All samples experienced the same storage period and were kept in airtight, odor-free containers under dry conditions to minimize volatile loss and oxidative changes.

### 2.2. Electronic Nose Analysis

Volatile odor profiles were recorded with a PEN3 portable electronic nose (AIRSENSE Analytics GmbH, Schwerin, Germany) equipped with 10 metal-oxide-semiconductor sensors (W1C, W5S, W3C, W6S, W5C, W1S, W1W, W2S, W2W, W3S), which exhibit broad and overlapping sensitivities to different volatile compound classes and were therefore interpreted collectively as a volatile fingerprint rather than as compound-specific detectors. Ground tea (1.0 g) was placed in a 20 mL headspace vial sealed with a PTFE/silicone septum (Agilent Technologies, Inc., Santa Clara, CA, USA), equilibrated at 50 °C for 30 min, and the headspace gas was drawn into the sensor chamber at 150 mL min^−1^ with clean dry air as the carrier. Sensor responses were recorded over a 60 s acquisition window after baseline stabilization, followed by a 60 s purge phase; the maximum response of each sensor was used for analysis. Electronic nose analysis followed established protocols for tea volatile profiling [28,31]. Each cultivar was analyzed in three independent replicates (*n* = 3).

### 2.3. Electronic-Tongue Analysis

Taste profiles were recorded with an SA-402B taste-analysis system (INSENT, Inc., Atsugi, Japan) using lipid-membrane potentiometric sensors. Infusions were prepared by extracting 1.0 g of ground tea in 100 mL deionized water (boiling-water bath, 15 min), cooling to room temperature, and centrifuging (3000 rpm, 10 min); supernatants were used as sample solutions. The instrument outputs eight channels—five initial tastes (sourness, bitterness, astringency, umami, saltiness) and three aftertastes (Aftertaste-A [astringent], Aftertaste-B [bitter], richness). These electronic-tongue outputs were treated as instrumental taste-related response signals and were not interpreted as direct measurements of human sensory perception. Sensors were conditioned and calibrated with the manufacturer’s standard solutions before each run [32,33]. The three technical measurements for each biological replicate were averaged to obtain a single value for each taste attribute. These biological-replicate means (*n* = 3 per cultivar), rather than the individual technical measurements, were used as independent observations in subsequent statistical analyses.

### 2.4. Headspace GC–MS Analysis of Volatile Metabolites

Volatile metabolites were profiled on an HS-15 headspace sampler (Panna Instruments, Beijing, China) coupled to a Trace GC Ultra gas chromatograph and a DSQ quadrupole mass spectrometer (Thermo Fisher Scientific, Waltham, MA, USA). Ground tea (2.0 g; NaCl-saturated (analytical grade, Sinopharm Chemical Reagent Co., Ltd., Shanghai, China)) was placed in a 20 mL headspace vial and equilibrated in the headspace oven at 50 °C for 15 min, with the sample loop maintained at 80 °C and the transfer line at 100 °C; vial pressurization was 20 s, loop-fill (quantification) time 3 s (time-based mode), and injection duration 3 min. Chromatographic separation was performed on a DB-5MS column (Agilent Technologies, Inc., Santa Clara, CA, USA; 30 m × 0.25 mm i.d., 0.25 μm film thickness) with helium as the carrier gas in constant-flow mode at 1.0 mL min^−1^. The injector was operated in PTV split mode at 180 °C with a split ratio of 10:1. The oven program was 60 °C (hold 3 min) → 150 °C at 5 °C min^−1^ → 230 °C at 10 °C min^−1^ (hold 5 min), giving a total run time of 38 min. Mass spectra were acquired in electron-impact mode (70 eV) in full-scan mode over *m*/*z* 35–350 at a scan rate of 500 amu s^−1^, with an ion-source temperature of 200 °C. Volatile annotations were based on mass-spectral matching against the NIST 20 library [34] (National Institute of Standards and Technology, Gaithersburg, MD, USA) together with linear retention indices calculated using a C7-C40 n-alkane series [35]. (Sigma-Aldrich, St. Louis, MO, USA). Features were retained when the NIST spectral similarity score was ≥800 and the difference between the experimental and reference retention indices was ≤30. Peaks detected in blank samples at 5 were considered background and excluded. Peak-area normalization was used for relative quantification. Raw data were processed (peak detection, deconvolution, alignment) in MS-DIAL (version 4.9; RIKEN Center for Sustainable Resource Science, Yokohama, Japan) [36]. There were three biological replicates per cultivar.

### 2.5. Untargeted UHPLC–QTOF-MS Metabolomics

Non-targeted profiling was performed on an Agilent 1290 Infinity II UHPLC (Agilent Technologies, Inc., Santa Clara, CA, USA) (Binary Pump G7120A, Multisampler G7167B, Column Compartment G7116B) coupled to a G6546A accurate-mass QTOF mass spectrometer (Agilent Technologies, Inc., Santa Clara, CA, USA) with a dual Agilent Jet Stream ESI source. Samples were prepared by ultrasonic extraction of 0.1 g ground tea in 1 mL 70% methanol (LC–MS grade, Merck KGaA, Darmstadt, Germany), containing internal standard (chloramphenicol; Sigma-Aldrich, St. Louis, MO, USA), centrifugation, and filtration (0.22 μm PTFE filter (MilliporeSigma, Burlington, MA, USA)). Separation was performed on a ZORBAX Eclipse Plus C18 RRHD column (Agilent Technologies, Inc., Santa Clara, CA, USA; 2.1 mm × 100 mm, 1.8 μm particle size) at 30 °C, with mobile phase (A) water + 0.1% formic acid (LC–MS grade, Fisher Scientific, Waltham, MA, USA) and (B) 95% acetonitrile (LC–MS grade, Merck KGaA, Darmstadt, Germany) in water, delivered at 0.300 mL min^−1^ with the following gradient: 0–19 min, 10.6% → 94.7% B; 19–20 min, hold 94.7% B; 20–21 min, 94.7% → 100% B; 21–22 min, hold 100% B; 22–24 min, 100% → 10.6% B; 24–25 min, re-equilibrate at 10.6% B. The injection volume was 0.10 μL and the total run time 26 min (MS data acquired during 1–20 min; diverter valve to waste outside this window).

MS detection was performed in both positive- and negative-ionization modes (separate runs) over *m*/*z* 100–1100 at a scan rate of 1.00 spectra s^−1^. The Dual AJS ESI source parameters were gas temperature 320 °C, drying-gas flow 8 L min^−1^, nebulizer pressure 35 psig, sheath-gas temperature 350 °C, sheath-gas flow 11 L min^−1^, nozzle voltage 1000 V, fragmentor 175 V, skimmer 65 V, octopole RF peak 750 V. The capillary voltage (VCap) was 4000 V in positive mode and 3500 V in negative mode. Real-time reference-mass correction was enabled (detection window 100 ppm, min height 1000 counts): ions 121.0509 and 922.0098 in positive mode; ions 112.9856 and 1033.9881 in negative mode. Acquisition thresholds were set at an absolute abundance of 200 (MS)/5 (MS/MS) and a relative threshold of 0.010%.

Metabolite annotation followed the Metabolomics Standards Initiative (MSI) framework [37]. Features were initially matched using accurate mass, isotope pattern, and MS1 information against METLIN (Scripps Research Institute, La Jolla, CA, USA) [38] and an in-house library. Metabolites matched to authentic reference standards analyzed under the same experimental conditions were classified as MSI Level 1 identifications. For selected high-abundance metabolites, Level 1 identification was accepted when the sample and authentic standard showed a retention-time relative deviation of <2%, co-eluted without peak splitting upon co-injection, had an accurate-mass error within 5 ppm, and exhibited an MS/MS cosine similarity of ≥0.8. Eight representative metabolites met all of these criteria. Features supported by accurate mass and diagnostic MS/MS fragmentation matched to library or database spectra, but without confirmation using an authentic standard, were classified as MSI Level 2 putative annotations. Accordingly, database MS/MS matches were not considered confirmed identifications. Detailed authentic-standard validation results are provided in Supplementary Table S6. Raw data were processed in Mass Profiler Professional (MPP, Agilent Technologies, Inc., Santa Clara, CA, USA; version B.15.1); features with RSD > 30% in pooled QC samples were removed. There were three biological replicates per cultivar.

### 2.6. Targeted LC–MS/MS Quantification of Flavonoids and Phenolic Compounds

Targeted quantification of 21 flavonoid and phenolic compounds—quinic acid, cinnamic acid, chlorogenic acid, caffeic acid, sinapic acid, coumalic acid, rosmarinic acid, salvianolic acid A, salvianolic acid B, p-coumaric acid, protocatechuic acid, caffeine, dihydromyricetin, vitexin, hesperidin, myricetrin, rutin, astragalin, quercetin, myricetin, and tangeretin—was performed on a Thermo Vanquish UHPLC system coupled to a TSQ Quantis triple-quadrupole mass spectrometer (Thermo Fisher Scientific, Waltham, MA, USA) in selected-reaction-monitoring (SRM) mode. Chromatographic separation used a C18 column (2.1 mm × 100 mm, 1.8 μm particle size; Thermo Hypersil Gold, Waltham, MA, USA) at 30 °C with mobile phase (A) 0.1% formic acid (LC–MS grade, Fisher Scientific, Waltham, MA, USA) (LC–MS grade, Fisher Scientific, Waltham, MA, USA) in water and (B) methanol (LC–MS grade, Merck KGaA, Darmstadt, Germany) (LC–MS grade, Merck KGaA, Darmstadt, Germany) at a flow rate of 0.300 mL min^−1^. The 5 min gradient was 0 min, 10% B; 3 min, 95% B (linear); 4 min, return to 10% B; 4–5 min, re-equilibration at 10% B. Ionization used a heated ESI (H-ESI) source in polarity-switching mode with a static spray voltage of 3500 V (positive)/3000 V (negative), sheath gas 50 (arbitrary units), auxiliary gas 10, sweep gas 1, ion-transfer-tube temperature 325 °C, and vaporizer temperature 350 °C. SRM acquisition used a cycle time of 0.6 s, Q1 and Q3 resolution 0.7 Da (FWHM), CID gas 1.5 mTorr, chromatographic peak width 6 s, and 10 points per peak. Two transitions (quantifier and qualifier) were monitored per compound with individually optimized collision energies (5.3–40.5 V) and RF-lens voltages (64–194 V); the full transition list is provided in Table S3. Authentic reference standards (purity ≥ 98%; Shanghai Yuanye Bio-Technology Co., Ltd., Shanghai, China) were used to confirm retention times and optimize MRM transitions; quantification used external calibration (r2 ≥ 0.99) with results expressed as μg kg^−1^ DW. There were three biological replicates per cultivar.

### 2.7. Physicochemical Quality Indices

Six physicochemical indices and DPPH (Sigma-Aldrich, St. Louis, MO, USA) radical-scavenging capacity were determined spectrophotometrically with a T10 UV-Vis spectrophotometer (Beijing Puxi General Instrument Co., Ltd., Beijing, China) on tea infusions prepared at a fixed tea-to-water ratio, with three biological replicates each. Polyphenol oxidase (PPO) activity was measured by homogenizing 1.0 g ground tea in pre-chilled phosphate buffer, using catechol as substrate (Sinopharm Chemical Reagent Co., Ltd., Shanghai, China) at 410 nm (one unit = 0.001 ΔA min^−1^ g^−1^ DW). Free amino acid content was determined by the ninhydrin method (Sigma-Aldrich, St. Louis, MO, USA; 570 nm; glutamic acid standard (Shanghai Yuanye Bio-Technology Co., Ltd., Shanghai, China); mg g^−1^ DW) [39]. Total phenolic content (TPC) was determined using the Folin–Ciocalteu method (Merck KGaA, Darmstadt, Germany; 765 nm; gallic acid equivalents [GAE Sigma-Aldrich, St. Louis, MO, USA], mg g^−1^ DW) [40]. Total flavonoid content (TFC) was determined using the NaNO_2_–AlCl_3_–NaOH method (analytical grade, Sinopharm Chemical Reagent Co., Ltd., Shanghai, China; 510 nm; rutin equivalents [RE, Shanghai Yuanye Bio-Technology Co., Ltd., Shanghai, China], mg g^−1^ DW) [41]. Soluble sugar content was determined by the anthrone–sulfuric acid method (Sinopharm Chemical Reagent Co., Ltd., Shanghai, China; 620 nm; glucose standard [Sinopharm Chemical Reagent Co., Ltd., Shanghai, China]; mg g^−1^ DW) [42]. Vitamin C was determined by the 2,6-dichloroindophenol method (Sigma-Aldrich, St. Louis, MO, USA; mg g^−1^ DW) [43]. DPPH scavenging capacity [44] was measured at 517 nm after 30 min incubation in the dark; results were expressed as percentage inhibition.

### 2.8. Experimental Design and Grouping

The study comprised eight cultivars × three biological replicates (*n* = 24 matched samples). Two platform-specific groupings were defined and are not interchangeable. For GC–MS aroma data, hierarchical cluster analysis (HCA) resolved three metabolotypes [45,46]: Group A (WZ, FHM, XSJ; three cultivars, *n* = 9 samples), Group B (YZH, JDX; two cultivars, *n* = 6 samples), and Group C (XG2, HBS, XG; three cultivars, *n* = 9 samples). For UHPLC–QTOF-MS data, PCA/OPLS-DA defined LC–MS Group A (FHM, HBS, XG) and LC–MS Group B (WZ, XSJ, JDX, XG2); YZH was unassigned. Because group membership is a property of cultivars, the cultivar, not the individual replicate, is the independent unit for comparisons between groups.

### 2.9. Statistical Analysis

All analyses were performed in R (version 4.3; R Foundation for Statistical Computing, Vienna, Austria) and Python (version 3.11; Python Software Foundation, Wilmington, DE, USA); significance was set at *p* < 0.05. For comparisons among cultivars, biological replicates (*n* = 3 per cultivar) were used as the independent statistical units, and cultivar effects were evaluated by one-way ANOVA with Duncan’s multiple range test [47] (e-nose sensors, e-tongue attributes, targeted compounds, physicochemical indices). PCA was performed on Z-score-normalized data; for the UHPLC–QTOF-MS dataset, positive, negative, and combined modes were computed separately. Pairwise OPLS-DA (Pareto scaling) [45,46] was applied to the three aroma metabolotype pairs (GC–MS) and to LC–MS Group A vs. Group B (both ion modes), after log_2_(x + 1) transformation and Pareto scaling. One predictive component and one (LC–MS) or two (GC–MS) automatically selected orthogonal components were used. Model fit was described by R^2^Y from the full-data model. Individual biological replicates were used as observational rows, whereas the cultivar was treated as the independent unit for validation and permutation. Validation was performed by leave-one-cultivar-out (LOCO) cross-validation, in which the three replicates of each cultivar were held out together and predicted from a model fitted to the remaining cultivars; LOCO Q^2^ was used as a diagnostic of the stability of group separation to the omission of individual cultivars, not as a measure of predictive performance for cultivars outside the studied set. Group separation was further assessed by an exact cultivar-level permutation test that enumerated all distinguishable assignments of group labels to cultivars (10 for the two-versus-three-cultivar comparisons and 35 for the three-versus-four-cultivar comparison; for the three-versus-three comparison, the 20 assignments form 10 mirror-image pairs that yield identical statistics). Under each assignment, the model was refitted and validated identically, and the exact *p* value was the proportion of assignments whose statistic was at least as large as that observed; the smallest attainable *p* values were therefore 0.10 for the GC–MS comparisons and 0.029 for the LC–MS comparison. CV-ANOVA was not applied because five to seven cultivar units provide inadequate residual degrees of freedom. All inferences from these models are restricted to the eight studied cultivars. Discriminant metabolites were prioritized using VIP > 1, FDR < 0.05, and |log_2_FC| ≥ 1 (≥2-fold change) as the primary stringent criterion. Because the univariate between-group tests (Welch’s *t*-test) treated biological replicates as observations, whereas group membership is defined at the cultivar level, the resulting *p* and FDR values were used as screening criteria for ranking features and not as the cultivar-level inference of group differences.

Cross-platform associations were assessed by Spearman rank correlation using individual biological replicates as the analytical unit (*n* = 24; eight cultivars × three replicates). Correlations were computed across all individual samples to describe associations among the analyzed materials, irrespective of whether variation arose between or within cultivars. Because replicates of a cultivar are not fully independent, replicate-level *p* and FDR values were used to screen and rank associations rather than as formal inference, and robustness was assessed by leave-one-cultivar-out direction consistency. A modular variable framework grouped 273 unique variables into 16 modules (2 e-nose, 4 e-tongue, 4 physicochemical/functional, and 6 metabolite-class); module scores were calculated as the equal-weighted mean of constituent Z-scores. Multiple-testing correction was performed using the Benjamini–Hochberg FDR procedure [48], applied separately within each domain. Robustness was evaluated by leave-one-cultivar-out (LOCO; 8-fold) cross-validation, recording direction consistency and the minimum, median, and maximum *p* values across the eight iterations. Significance tiers were defined as robust_strong (|ρ| ≥ 0.75, FDR < 0.05, LOCO ≥ 7/8) and exploratory (|ρ| ≥ 0.70, *p* < 0.05, LOCO ≥ 6/8).

Taste-trait associations were modeled by sparse partial least squares (sPLS) [49] and ElasticNet regression [50] on a de-redundant 31-variable predictor set (Tier 1/2 non-volatile metabolites, targeted analytes, TPC, TFC, amino acids, soluble sugars, vitamin C, PPO activity), validated by LOCO and 999-permutation testing, in which the responses of the three replicates of each cultivar were permuted together as a block, so that the cultivar was the exchangeable unit (reported as sPLS Q^2^ and *p*; *p* ≤ 0.001 denotes that no permutation reached the observed value). Ninety-one metabolite/quality-index ratios were tested: self-referential ratios were relabeled as efficiency indices (e.g., DPPH per TPC/TFC), and non-self-referential ratios retained as balance indices. Antioxidant candidates were prioritized by integrating 54 univariate linear-regression models with sPLS, Ridge, and TPC-only multivariate models plus permutation testing. Ten pathway/product-class scores (mean Z-score of class members) were compared across groups descriptively, using η^2^ (aroma groups) or Cohen’s d (LC–MS groups) as effect sizes, and marker direction consistency was assessed by binomial tests. Across all domains, 2012 association pairs met the nominal significance threshold (*p* < 0.05) and were subsequently graded according to FDR support, effect size, and LOCO robustness into Tier 1 (53), Tier 2 (57), Tier 3 (1038), and Excluded (864). Tier 1 comprised FDR-supported robust associations (FDR < 0.05, |ρ| ≥ 0.75, LOCO ≥ 7/8), whereas Tier 2 represented robust exploratory associations supported by nominal significance and LOCO consistency. Thus, statistical significance alone was not interpreted as evidence of biological importance; biological prioritization additionally required effect-size magnitude and cross-validation robustness.

GC–MS data were processed in MS-DIAL (version 4.9; RIKEN Center for Sustainable Resource Science, Yokohama, Japan) and annotated against the NIST 20 library (National Institute of Standards and Technology, Gaithersburg, MD, USA); UHPLC–QTOF-MS data were processed in Mass Profiler Professional (MPP; Agilent Technologies, Inc., Santa Clara, CA, USA; version B.15.1) and annotated against METLIN (Scripps Research Institute, La Jolla, CA, USA) and an in-house library. R packages used included mixOmics (v 6.22; Bioconductor), glmnet (v 4.1; CRAN), and tidyverse (v 2.0; CRAN); Python packages included scikit-learn (v 1.3; scikit-learn developers) and SciPy (v 1.11; SciPy developers).

## 3. Results

### 3.1. Electronic Nose Analysis of Volatile Odor Profiles Among Guava Tea Cultivars

To characterize the overall volatile odor fingerprints of the eight guava cultivars, electronic nose (e-nose) analysis was performed using a 10-sensor array. One-way ANOVA revealed significant differences among cultivars across all 10 sensors (Table 1), with six sensors—W1C, W3C, W3S, W6S, W5C, and W2S—reaching highly significant levels (*p* < 0.001). The highest F-values were recorded for W1C (F = 26.05) and W3C (F = 23.95), indicating that variation in these two sensor responses contributed strongly to cultivar discrimination.

An examination of sensor response profiles revealed two contrasting volatile response types: ‘Yanzhi Hong’ exhibited the highest responses in W5S, W6S, W1S, W1W, W2S, W2W, and W3S, whereas Hongbaoshi showed the highest responses in W1C, W3C, and W5C. These contrasting sensor-response patterns indicate distinct volatile fingerprints between the two cultivars, but do not by themselves establish enrichment of specific chemical classes.

**Figure 1:**
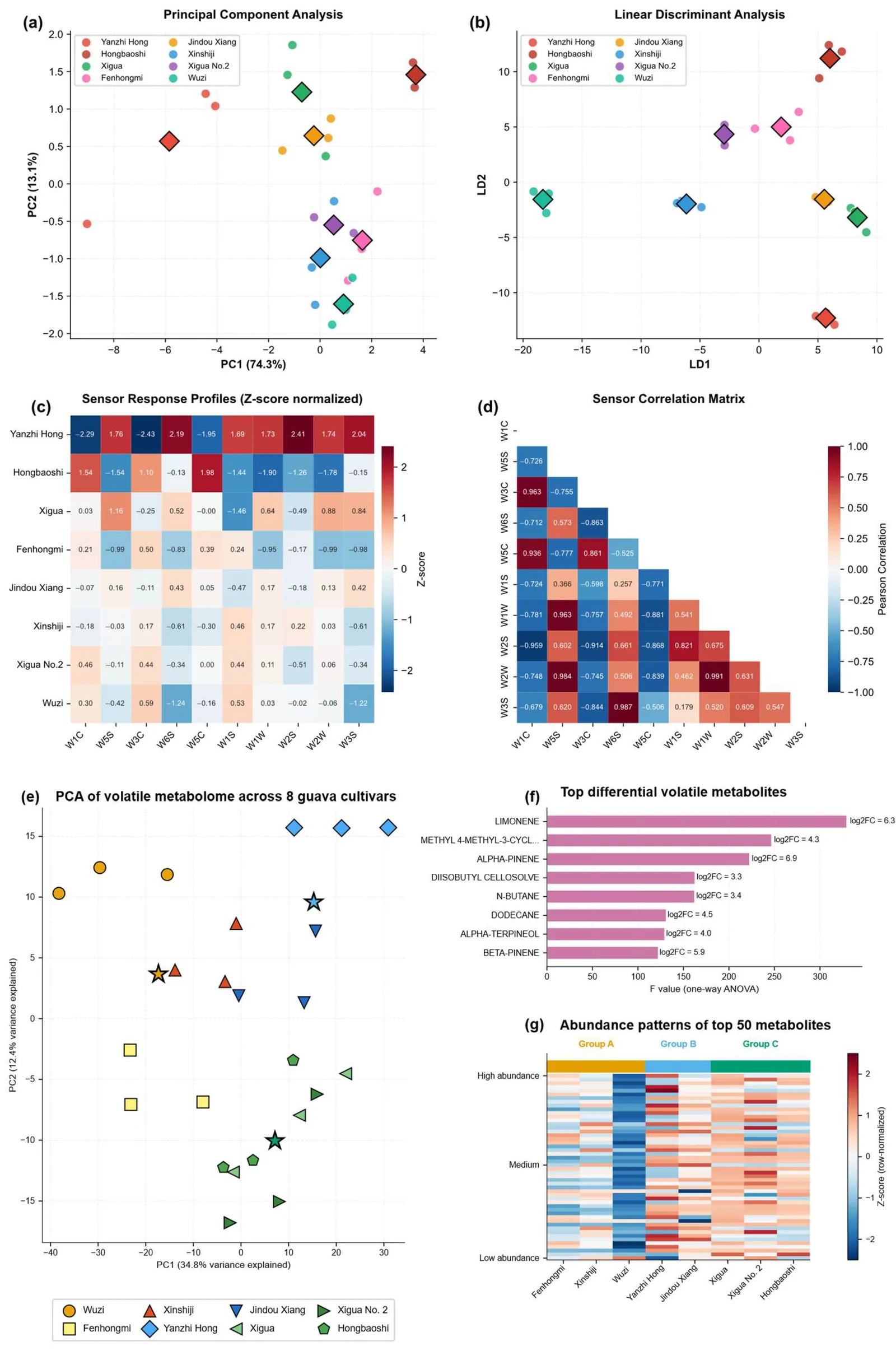
Distinct volatile odor fingerprints of eight guava cultivars detected by electronic nose (**a**–**d**), and characterization of volatile metabolome profiles of guava leaf tea from eight cultivars by HS-GC–MS (**e**–**g**). (**a**) PCA score plot showing separation of cultivars along PC1 and PC2, accounting for 87.4% cumulative variance. Circles represent biological replicates; diamonds indicate cultivar centroids. (**b**) LDA score plot demonstrating cultivar-level discrimination capacity of the e-nose system. (**c**) Z-score-normalized heatmap of sensor response profiles, revealing contrasting volatile enrichment patterns between Yanzhi Hong and Hongbaoshi. (**d**) Sensor correlation matrix identifying two mutually opposed response modules that underpin cultivar differentiation. (**e**) Principal component analysis (PCA) score plot showing separation of volatile metabolomes among 8 guava cultivars. PC1 and PC2 explain 34.8% and 12.4% of total variance, respectively. Each cultivar is represented by distinct colors and shapes; stars indicate group centroids, with each star color corresponding to the respective cultivar. (**f**) Bar plot of ANOVA F-values for the top-8 most significantly differential volatile metabolites. Numbers to the right of bars indicate maximum log2 fold-change (log2FC) across cultivars. (**g**) Heatmap of abundance patterns for the top-50 differential metabolites. Columns represent 8 cultivars grouped by hierarchical clustering. Color bars above indicate group membership: Group A (orange), Group B (blue), Group C (green). Color gradient represents Z-score-normalized relative abundance (red = high, blue = low).

‘Xigua’ shared partial similarity with ‘Yanzhi Hong’ in W5S, W1W, W2W, W3S, and W6S, while the remaining cultivars—‘Fenhongmi’, ‘JindouXiang’, ‘Xinshiji’, ‘Xigua No. 2′, and ‘Wuzi’—displayed intermediate response patterns (Figure 1b,c).

Principal component analysis (PCA) further resolved the volatile odor profiles of the eight cultivars. PC1 and PC2 accounted for 74.31% and 13.13% of the total variance, respectively (cumulative: 87.44%), indicating that the major variation was captured by the first two components (Figure 1a). PCA loading analysis showed that PC1 was positively driven by W1C, W3C, and W5C (loadings: 0.352, 0.355, and 0.342), and negatively driven by W2S, W1W, W2W, W5S, W6S, and W3S (loadings: −0.330, −0.327, −0.321, −0.317, −0.279, and −0.276), showing that PC1 primarily separates two opposing groups of cross-sensitive sensor responses. Along PC1, ‘Yanzhi Hong’ (mean score: −5.85) and ‘Hongbaoshi’ (mean score: 3.71) occupied opposite extremes, further demonstrating their contrasting volatile phenotypes.

Linear discriminant analysis (LDA) provided a supervised visualization of cultivar-associated differences in the e-nose response patterns. In LDA space, ‘Yanzhi Hong’ and ‘Hongbaoshi’ were clearly separated along LD2 (mean LD2 scores: −12.29 and 11.21, respectively), while ‘Wuzi’ showed the most negative LD1 score, indicating a distinct volatile signature from the remaining cultivars (Figure 1a,b).

Correlation analysis among the 10 sensors revealed two internally coherent but mutually opposing response modules (Figure 1d). W1C, W3C, and W5C were strongly positively correlated (W1C–W3C: r = 0.963; W1C–W5C: r = 0.936), whereas W5S, W1W, W2W, W2S, W6S, and W3S formed a second highly correlated module (W1W–W2W: r = 0.991; W6S–W3S: r = 0.987). Strong negative cross-module correlations were consistently observed (e.g., W1C–W2S: r = −0.959; W3C–W6S: r = −0.864), indicating that cultivar-level volatile differentiation reflects a restructuring of odor response spectra rather than a uniform shift in overall volatile intensity. Collectively, these results demonstrate that the eight guava cultivars possess distinct and reproducible volatile odor fingerprints detectable by e-nose, providing phenotypic evidence for further characterization of their volatile compound compositions.

### 3.2. Volatile Metabolic Profiling by HS-GC–MS Reveals Three Aroma Metabolotypes Across Guava Leaf Tea Cultivars

To characterize volatile aroma metabolite diversity across leaf tea from eight guava cultivars, we first performed PCA on headspace metabolomics data (Figure 1e). The score plot revealed a clear cultivar-dependent distribution, with PC1 and PC2 jointly explaining approximately 47.2% of total variance. ‘Wuzi’, ‘Fenhongmi’, and ‘Xinshiji’ clustered in the negative PC1 region; ‘Yanzhi Hong’ and ‘Jindou Xiang’ shifted toward positive PC2; while ‘Xigua No. 2′, ‘Hongbaoshi’, and ‘Xigua’ grouped tightly in the negative PC2 region, indicating highly similar volatile profiles. ‘Wuzi’, ‘Fenhongmi’, and ‘Xinshiji’ showed greater within-group dispersion, particularly along PC2, suggesting intra-group metabolic heterogeneity.

One-way ANOVA identified 482 significantly differential volatile metabolites across cultivars (all FDR < 0.05) (Figure 1f). Compounds including limonene, methyl 4-methyl-3-cyclohexenyl ketone, alpha-pinene, beta-pinene, and alpha-terpineol exhibited the highest F-values, with log2FC values ranging from approximately 1.01 to 7.42. HCA based on these 482 metabolites consistently classified the eight cultivars into three major volatile metabolic groups (Figure 1g): Group A (‘Wuzi’, ‘Fenhongmi’, ‘Xinshiji’), Group B (‘Yanzhi Hong’, ‘Jindou Xiang’), and Group C (‘Xigua No. 2′, ‘Hongbaoshi’, ‘Xigua’). This tripartite classification was well-supported by PCA, with Group C showing the tightest clustering in both analyses.

Comparison of mean abundance profiles across the three groups revealed markedly asymmetric patterns (Figure 1g). Group B showed the highest abundance for 296 of the 482 differential metabolites, Group C for 168, and Group A for only 18. Conversely, Group A exhibited the lowest abundance for 397 metabolites, indicating that, at the group-mean level, ‘Wuzi’, ‘Fenhongmi’, and ‘Xinshiji’ are characterized by a lower abundance of most differential volatiles rather than by the selective enrichment of group-specific compounds; the total volatile abundance of these cultivars nevertheless overlapped with that of Group C cultivars. Group B was enriched in monoterpenes and their derivatives—including limonene, alpha-pinene, beta-pinene, myrtenyl acetate, isoborneol, and camphene—consistent with a terpene-dominant aroma signature. Group C was distinguished by higher levels of cis-nerolidyl acetate, methyl 4-methyl-3-cyclohexenyl ketone, eicosane, ortho-cymene, alpha-E-ionol, and related compounds, pointing to a sesquiterpene ester-, alkane-, and ketone-associated volatile background.

**Figure 2:**
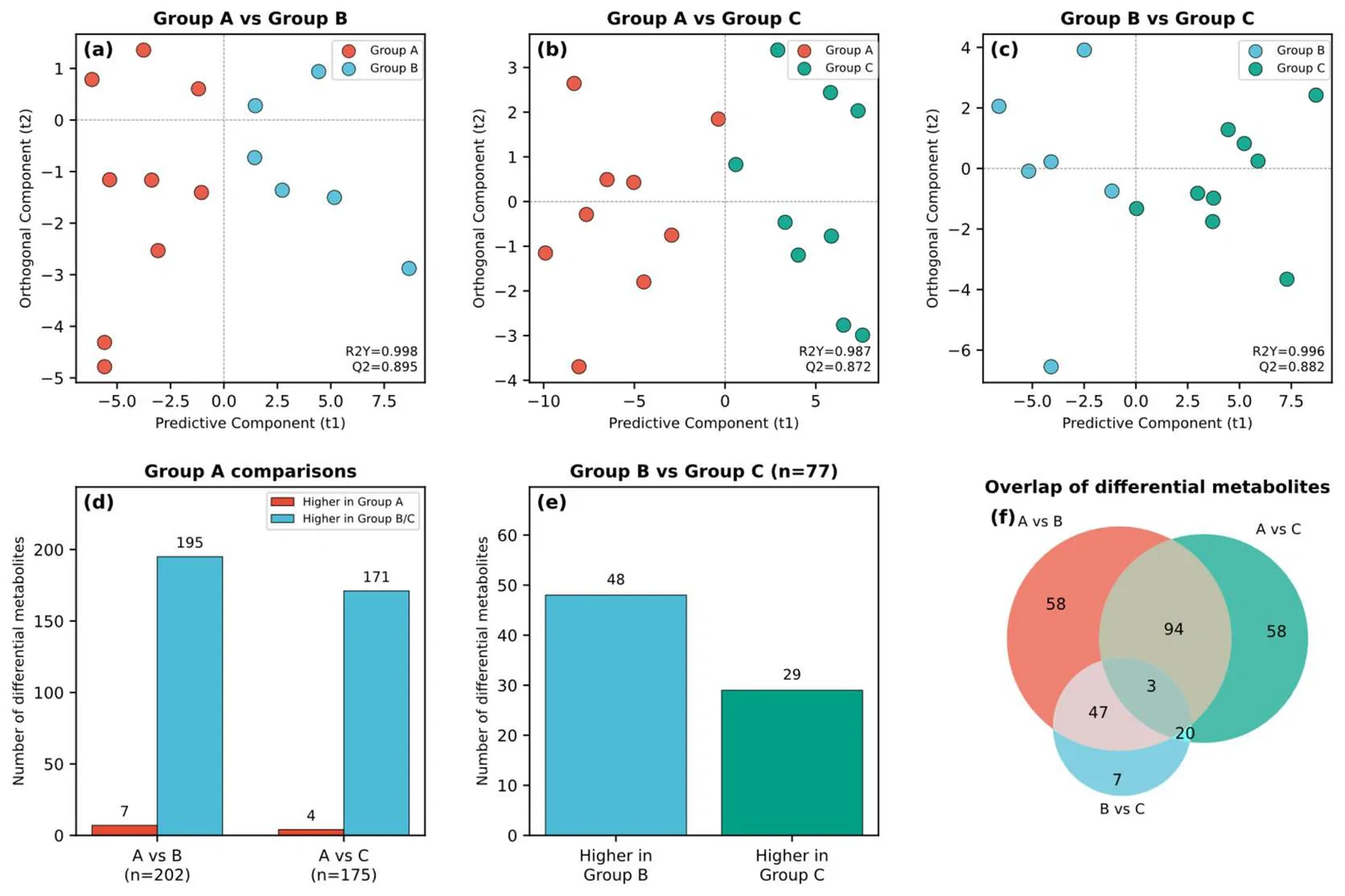
Pairwise OPLS-DA analysis prioritizes discriminant volatile metabolites among three metabolic phenotypes (**a**–**c**). OPLS-DA score plots showing clear separation between each pair of comparison groups: (**a**) Group A vs. Group B, (**b**) Group A vs. Group C, and (**c**) Group B vs. Group C. R^2^Y values refer to the full-data fit; cultivar-level validation results (LOCO Q^2^ and exact permutation *p* values) are given in Table S1. (**d**) Bar chart summarizing the number of differential metabolites in Group A comparisons, showing the majority of differential metabolites are more abundant in Group B (A vs. B, *n* = 202) or Group C (A vs. C, *n* = 175), consistent with lower abundance of most discriminant volatiles in Group A. (**e**) Bar chart for Group B vs. Group C comparison (*n* = 77 differential metabolites), revealing distinct compositional differences with each group containing specifically enriched compounds. (**f**) Venn diagram illustrating the overlap of candidate differential metabolites across all three pairwise comparisons. Different colors represent different pairwise comparisons: red, Group A vs. Group B; green, Group A vs. Group C; and blue, Group B vs. Group C. In total, 355 unique discriminant candidates were prioritized across all comparisons.

To further resolve the metabolites contributing to inter-group divergence, pairwise OPLS-DA was conducted for all three group combinations (Figure 2a–c). Replicates of each cultivar clustered together, and the groups were separated along the predictive component in all score plots. Group A vs. Group B and Group A vs. Group C yielded 202 and 175 prioritized discriminant metabolites (meeting the VIP, fold-change, *p* and FDR screening thresholds), respectively (Figure 2d). In both comparisons, the large majority of these metabolites were more abundant in Group B or Group C, consistent with lower abundance of most discriminant volatiles in Group A.

Model validation was performed at the cultivar level (Table S1). The three GC–MS models fitted the data closely (R^2^Y = 0.988–0.998); however, because R^2^Y approaches 1 for high-dimensional data largely irrespective of group structure, it was not used as evidence of separation. Exact cultivar-level permutation *p* values for Q^2^ were 0.20 (A vs. B), 0.10 (A vs. C) and 0.10 (B vs. C); with five or six cultivars, only ten distinct cultivar partitions exist per comparison, so *p* values below 0.10 cannot be attained. LOCO Q^2^ was 0.317 for A vs. C but negative for A vs. B (−0.307) and B vs. C (−0.200), indicating that these separations depend on particular cultivars rather than being expressed uniformly by every cultivar within a group. The pairwise GC–MS models are therefore used descriptively, to rank the features contributing to the observed differences among the studied cultivars, rather than as statistical confirmation of the three metabolotypes.

The Group B vs. Group C comparison yielded 77 prioritized discriminant metabolites, with each group contributing group-specifically enriched compounds (Figure 2e), reflecting compositional rather than purely quantitative differences between two high-abundance metabolotypes. Group B-enriched candidates included alpha-pinene, limonene, beta-pinene, myrtenyl acetate, isoborneol, alpha-fenchyl acetate, camphene, and alpha-terpinene, with alpha-pinene, limonene, and beta-pinene displaying the highest VIP scores (1.923, 1.922 and 1.917; Table S4). Group C-enriched candidates included cis-nerolidyl acetate, sesquisabinene, beta-butyrolactone, and methyl 4-methyl-3-cyclohexenyl ketone. Across all three comparisons, 355 unique discriminant candidates were prioritized (Figure 2f). Collectively, these results indicate that aroma divergence among the studied cultivars involves both shifts in overall volatile abundance patterns and differences in the relative abundance of distinct compound classes. The three metabolotypes—terpene-dominant (Group B), sesquiterpene ester-/alkane-/ketone-associated (Group C), and characterized by lower abundance of most discriminant volatiles (Group A)—describe the volatile composition patterns observed among the eight cultivars.

Among the 77 strict differential volatile metabolites detected between Group B and Group C, 47 were enriched in Group B and 26 in Group C after excluding low-confidence/background features and counting co-eluting duplicate annotations only once (Table S4). Group B-enriched metabolites were strongly dominated by monoterpenes and related derivatives, which accounted for 15 of the 47 enriched metabolites and 96.4% of their combined mean abundance. By contrast, Group C-enriched metabolites spanned 11 chemical classes, indicating broader compositional diversity, although abundance remained concentrated in a limited subset of compounds. In Group C, cis-nerolidyl acetate was the single largest contributor, accounting for 48.4% of the total mean abundance of Group C-enriched metabolites, followed by alkane- and ketone-associated components (see Supplementary Methods).

### 3.3. Electronic-Tongue-Based Instrumental Taste-Response Profiling of Eight Guava Tea Cultivars

One-way ANOVA demonstrated that all eight electronic-tongue taste attributes differed highly significantly among the eight guava tea cultivars (*p* < 0.001 for all attributes; Table 2). Saltiness showed the highest discriminatory power (F = 995.42), followed by astringency (F = 704.85) and Aftertaste-B (F = 435.80), indicating that the corresponding instrumental responses contributed most strongly to cultivar-level differentiation.

**Figure 3:**
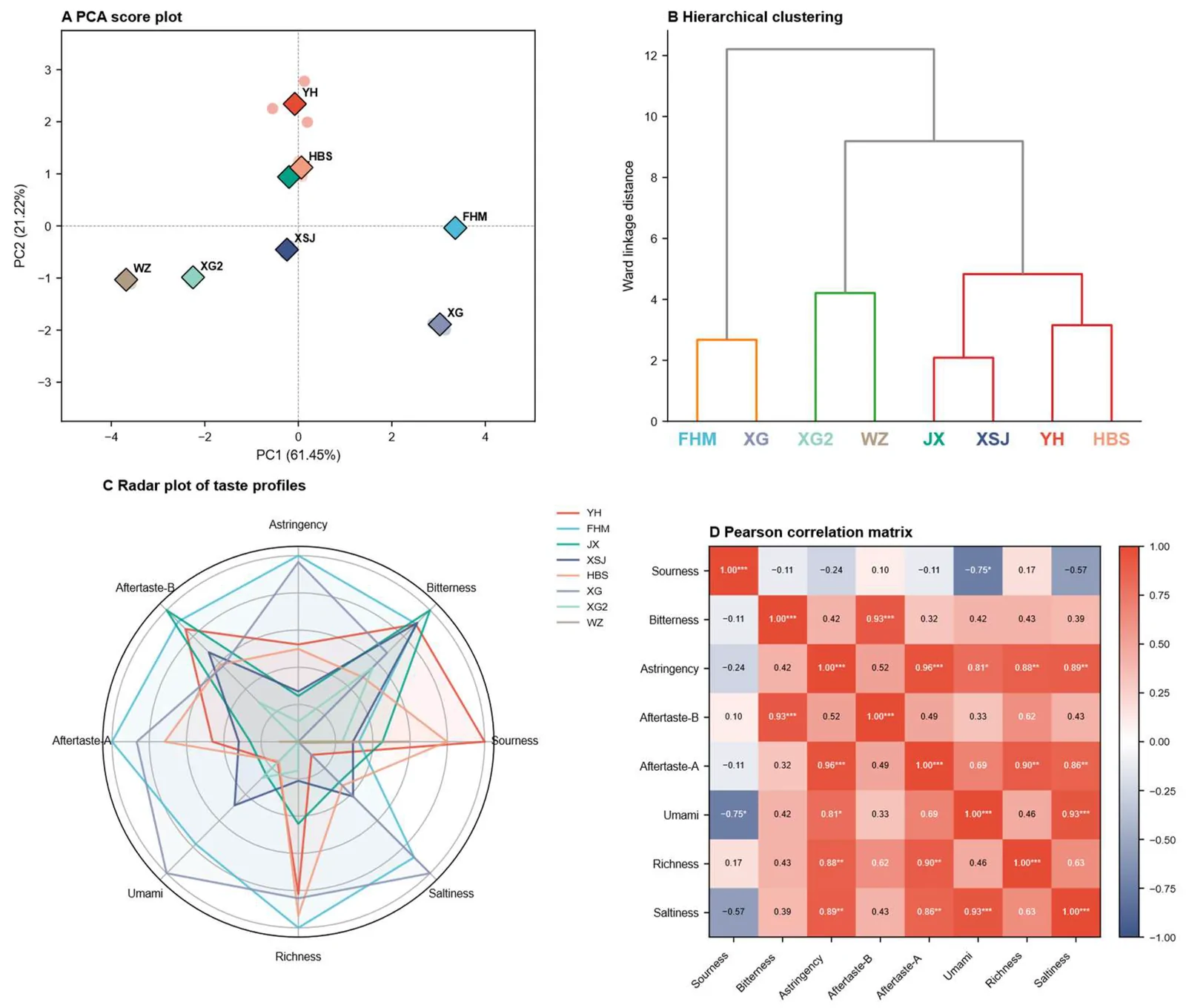
Instrumental taste-related response profiles of eight guava tea cultivars measured using electronic-tongue analysis. (**A**) Principal component analysis (PCA) score plot based on eight taste attributes. The first two principal components collectively explain 82.67% of the total variance. Different colors indicate different cultivars. Semi-transparent symbols represent individual biological replicates (*n* = 3 per cultivar), while solid symbols with black borders denote cultivar means. (**B**) Hierarchical clustering dendrogram based on Euclidean distances and Ward’s method, showing similarity relationships among cultivar mean taste profiles. Colored lines distinguish clusters, whereas gray lines indicate higher-level linkages between clusters. (**C**) Radar plot displaying normalized taste profiles across eight attributes: sourness, bitterness, astringency, Aftertaste-B, Aftertaste-A, umami, richness, and saltiness. Each colored line represents one guava cultivar. (**D**) Pearson correlation heatmap of the eight taste attributes. Correlation coefficients (r) are indicated within each cell, with color intensity ranging from blue (negative correlation) to red (positive correlation). Asterisks indicate statistical significance: * *p* < 0.05, ** *p* < 0.01, and *** *p* < 0.001.

The remaining attributes—Aftertaste-A, bitterness, sourness, umami, and richness—also exhibited highly significant inter-cultivar variation, demonstrating that electronic-tongue response differences among guava cultivars are multidimensional rather than confined to a single instrumental response axis. The taste profiles of individual cultivars, visualized across all eight attributes, are presented in Figure 3C.

Radar plot analysis revealed distinct taste profile shapes among cultivars (Figure 3C). ‘Fenhongmi’ and ‘Xigua’ displayed the largest overall polygon areas, driven by elevated astringency, Aftertaste-A, umami, and saltiness. ‘Yanzhi Hong’ and ‘Jindou Xiang’ showed prominent bitterness and Aftertaste-B responses. ‘Wuzi’ and ‘Xigua No. 2′ exhibited the smallest polygon areas across most attributes, consistent with their generally lower electronic-tongue response intensities. ‘Hongbaoshi’ showed a relatively distinct profile characterized by higher sourness and richness compared to other cultivars, while ‘Xinshiji’ displayed a moderate and relatively balanced electronic-tongue response profile without a dominant response dimension. To characterize the overall separation pattern among cultivars, PCA was performed on Z-score-normalized data from all eight attributes (Figure 3A). The first two principal components collectively accounted for 82.67% of total variance (PC1: 61.45%; PC2: 21.22%). PC1 was predominantly loaded by astringency (0.431), saltiness (0.414), Aftertaste-A (0.407), umami (0.385), and richness (0.370), representing a composite axis of astringency, umami–saltiness, and taste richness. PC2 was most strongly associated with sourness (0.621), Aftertaste-B (0.457), and bitterness (0.334), with negative loadings for umami (−0.374) and saltiness (−0.243), reflecting a sourness-and-bitterness axis inversely related to umami–saltiness intensity. Along PC1, ‘Fenhongmi’ (PC1 = 3.341) and ‘Xigua’ (PC1 = 3.100) were positioned in the strongly positive direction, consistent with their elevated astringency, umami, and saltiness responses, whereas ‘Wuzi’ (PC1 = −3.725) and ‘Xigua No. 2′ (PC1 = −2.231) were located in the negative direction, indicating relatively low overall taste intensity. Along PC2, ‘Yanzhi Hong’ (PC2 = 2.178), ‘Hongbaoshi’ (PC2 = 1.287), and ‘Jindou Xiang’ (PC2 = 1.025) were distributed toward the positive direction; among these, ‘Yanzhi Hong’ and ‘Jindou Xiang’ were mainly distinguished by elevated bitterness and Aftertaste-B, while ‘Hongbaoshi’ was characterized by higher sourness and richness. ‘Xinshiji’ was located near the centroid of the score plot, with no single taste attribute predominating.

Hierarchical clustering analysis based on cultivar-mean taste profiles further corroborated the PCA groupings (Figure 3B). ‘Fenhongmi’ and ‘Xigua’ clustered into an adjacent branch, reflecting their shared high-astringency and high-umami–saltiness characteristics, whereas ‘Wuzi’ and ‘Xigua No. 2′ formed a distinct low-intensity cluster. ‘Yanzhi Hong’, ‘Hongbaoshi’, ‘Jindou Xiang’, and ‘Xinshiji’ were resolved into a separate major branch; within this group, ‘Yanzhi Hong’ and ‘Hongbaoshi’ were more closely associated, while ‘Jindou Xiang’ and ‘Xinshiji’ showed a proximal but internally heterogeneous relationship.

Pearson correlation analysis revealed three coordinated taste-response modules among the eight attributes (Figure 3D). Within the astringency–aftertaste module, astringency and Aftertaste-A were very strongly correlated (r = 0.956, *p* = 3.16 × 10^−13^). Within the umami–saltiness module, umami and saltiness co-varied strongly across cultivars (r = 0.929, *p* = 5.34 × 10^−11^). Within the bitterness module, bitterness and Aftertaste-B were also highly correlated (r = 0.929, *p* = 5.72 × 10^−11^). In addition, astringency was significantly correlated with umami (r = 0.815, *p* = 1.26 × 10^−6^), indicating that cultivars with stronger astringency tended to also display higher umami responses, consistent with the profiles of ‘Fenhongmi’ and ‘Xigua’. Because electronic-tongue sensors can exhibit partially overlapping cross-responses, this correlation should be interpreted as covariance of instrumental responses across cultivars rather than evidence of a shared chemical basis for astringency and umami. Sourness was negatively correlated with both umami (r = −0.786, *p* = 5.22 × 10^−6^) and saltiness (r = −0.578, *p* = 0.003), suggesting an opposing relationship between sourness-related and umami–saltiness-related responses across cultivars.

### 3.4. Non-Targeted Metabolomics Reveals Cultivar-Level Metabolic Divergence Among Eight Guava Cultivars: A Comparative Analysis

**Figure 4:**
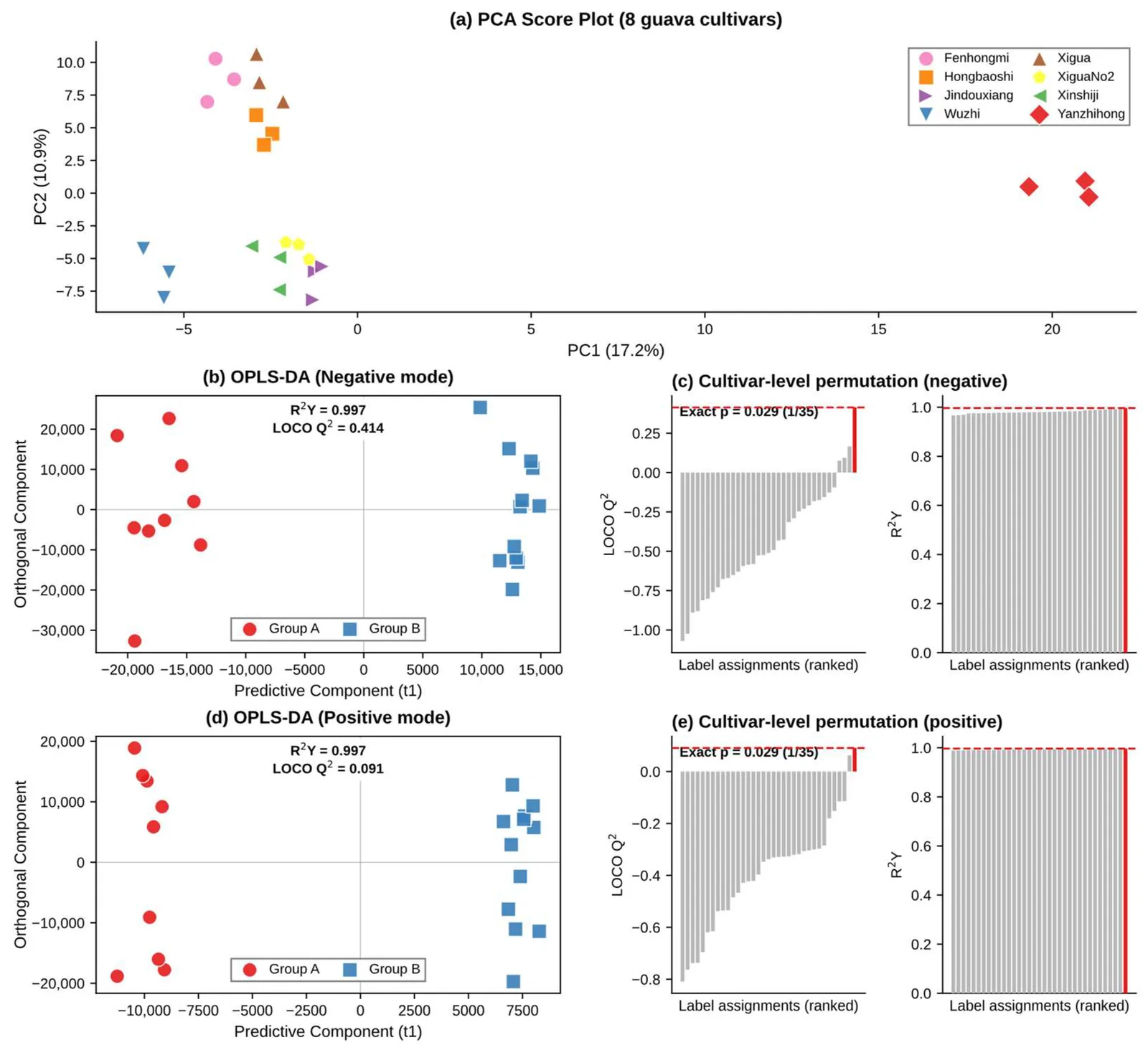
Multivariate statistical analysis of metabolic profiles in guava tea leaves. (**a**) Principal component analysis (PCA) score plot of metabolite profiles from 8 guava cultivars based on combined positive- and negative-ion mode data. The variance explained by PC1 and PC2 is indicated in parentheses. Each cultivar is represented by a distinct color and marker as shown in the legend. (**b**) Orthogonal partial least squares discriminant analysis (OPLS-DA) score plot for Group A vs. Group B in negative-ion mode. Group A includes ‘Fenhongmi’, ‘Hongbaoshi’, and ‘Xigua’; Group B includes ‘Wuzi’, ‘Xinshiji’, ‘Jindou Xiang’, and ‘XiguaNo2′. R^2^Y = 0.997 (full-data fit); LOCO Q^2^ = 0.414. Red circles = Group A; blue squares = Group B; the grey lines are value markers. (**c**) Exact cultivar-level permutation test for the negative-ion OPLS-DA model. Grey Bars show LOCO Q^2^ (left) and R^2^Y (right) for all 35 distinguishable assignments of group labels to the seven cultivars; the red bar marks the observed grouping, whose statistic is the largest of all 35 assignments (exact *p* = 1/35 = 0.029), and the dashed line indicates its value. (**d**) OPLS-DA score plot for Group A vs. Group B in positive-ion mode. R^2^Y = 0.997 (full-data fit); LOCO Q^2^ = 0.091. Red circles = Group A; blue squares = Group B; the grey lines are value markers. (**e**) Exact cultivar-level permutation test for the positive-ion OPLS-DA model, displayed as in (**c**). Exact *p* = 0.029 for both statistics.

Non-targeted UHPLC–QTOF-MS metabolomics was performed on guava tea prepared from eight cultivars—‘Xigua’, ‘Xigua No. 2′, ‘Yanzhi Hong’, ‘Jindou Xiang’, ‘Xinshiji’, ‘Hongbaoshi’, ‘Wuzi’, and ‘Fenhongmi’—to characterize their overall metabolic diversity and identify cultivar-discriminating metabolites. PCA was conducted separately on positive-ion mode, negative-ion mode, and the combined dataset as an unsupervised overview of inter-cultivar metabolic variation (Figure 4a). The first two principal components explained 17.2% and 10.9% of total variance, respectively, and score plots revealed that the eight cultivars occupied distinct regions of the PCA space. Notably, ‘Yanzhi Hong’ was markedly separated from the remaining seven cultivars along PC1, occupying a remote position in the PCA space that was not shared by any other cultivar, suggesting a substantially divergent overall metabolic composition. Among the remaining seven cultivars, score plots indicated two broad groupings: ‘Xigua’, ‘Hongbaoshi’, and ‘Fenhongmi’ clustered together and were designated Group A, while ‘Xigua No. 2′, ‘Jindou Xiang’, ‘Xinshiji’, and ‘Wuzi’ formed a second cluster designated Group B.

To characterize the metabolic differences underlying these groupings, OPLS-DA models were constructed for two pairwise comparisons: ‘Yanzhi Hong’ versus all other cultivars, and Group A versus Group B. The ‘Yanzhi Hong’ versus others comparison did not yield a validated model in either ion mode, indicating that, despite its pronounced displacement in the PCA space, ‘Yanzhi Hong’ does not form a statistically discriminable metabolic cluster relative to the remaining cultivars when quantitative peak area variables are used exclusively; this comparison was therefore not pursued further. In contrast, the Group A versus Group B comparison fitted the data closely in both negative (R^2^Y = 0.997; Figure 4b) and positive-ion mode (R^2^Y = 0.997; Figure 4d). In the exact cultivar-level permutation test, which enumerated all 35 distinguishable assignments of the seven cultivars to a three-versus-four design, the observed assignment produced the largest Q^2^ and R^2^Y in both ion modes (*p* = 0.029, the smallest attainable value; Figure 4c,e; Table S1). LOCO Q^2^ was 0.414 in negative-ion mode and 0.091 in positive-ion mode, indicating that the negative-ion separation was stable to the omission of individual cultivars, whereas the positive-ion separation depended more strongly on particular cultivars. Within the seven cultivars compared, the metabolic divergence between Group A (‘Xigua’, ‘Hongbaoshi’, ‘Fenhongmi’) and Group B (‘Xigua No. 2′, ‘Jindou Xiang’, ‘Xinshiji’, ‘Wuzi’) is therefore supported primarily by the negative-ion data, with consistent but weaker support from the positive-ion data.

**Figure 5:**
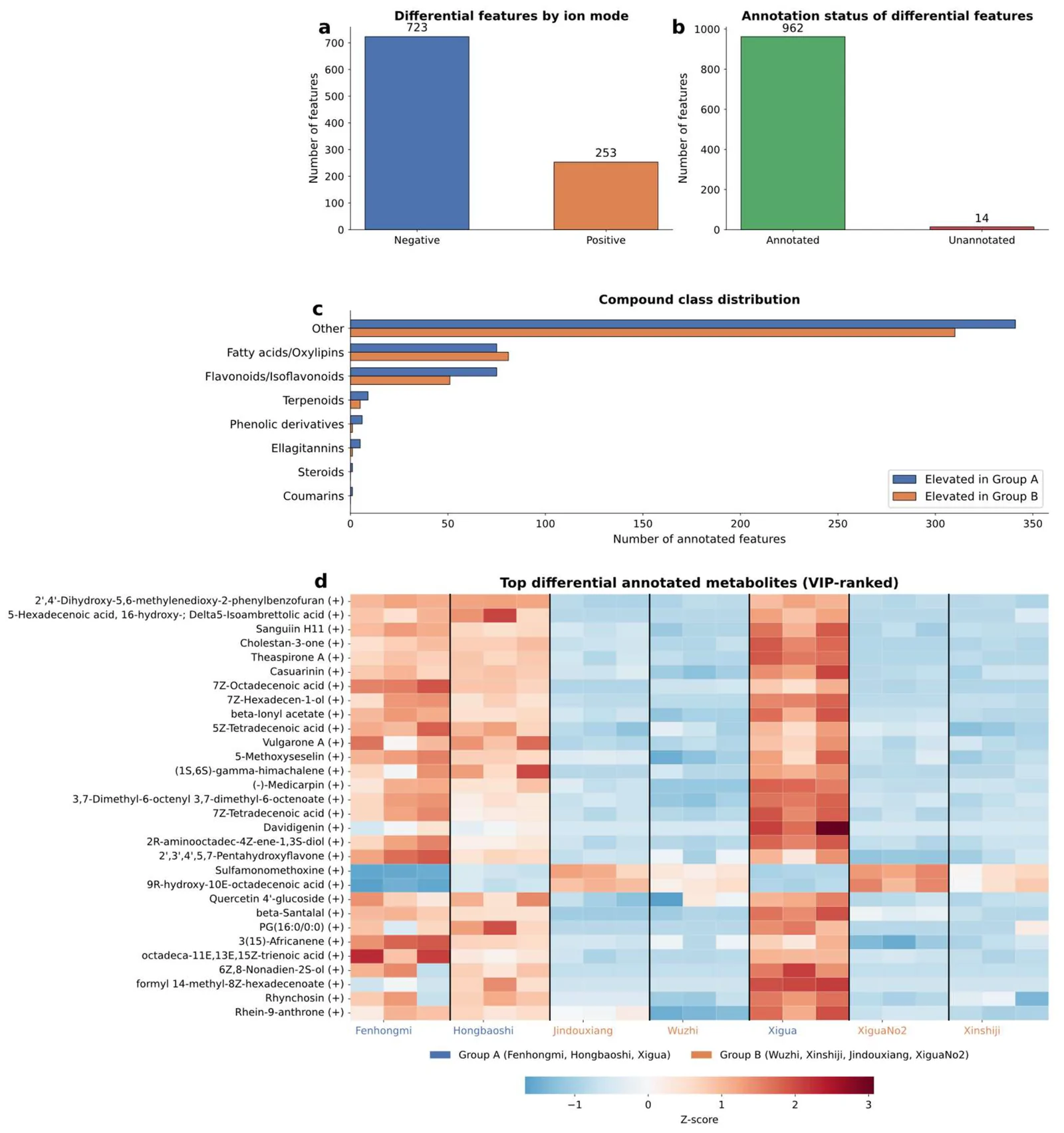
Overview of prioritized discriminant features between Group A and Group B guava cultivars. (**a**) Number of differential features elevated (349) or reduced (224) in Group A relative to Group B after applying the final screening criteria (VIP > 1, |log_2_FC| ≥ 1, FDR < 0.05; replicates treated as observations). (**b**) Annotation status of the 573 final differential features, including 566 annotated and 7 unannotated features. (**c**) Distribution of compound classes among annotated differential features, showing counts elevated in Group A (blue) and Group B (orange). (**d**) Heatmap of the top-30 annotated discriminant features ranked by VIP score. Columns represent individual samples, with blue/orange labels indicating Group A/B. Black vertical lines separate different cultivars. Color intensity represents Z-score-normalized relative abundance.

Features contributing to Group A/Group B discrimination were first extracted using VIP > 1, yielding 2127 features in negative-ion mode and 1211 features in positive-ion mode (3338 in total). Applying fold-change (FC ≥ 2 or ≤ 0.5, Group A/Group B) and nominal *p* < 0.05 thresholds retained 976 candidate features, and, after Benjamini–Hochberg correction across all tested features, 573 features met the final screening criterion (VIP > 1, |log_2_FC| ≥ 1 and FDR < 0.05) (Figure 5a). As described in Section 2.9, these *p* and FDR values treat replicates as observations and were used to prioritize discriminant features rather than being used as cultivar-level tests of group differences. Of these 573 differential features, 349 were elevated and 224 were reduced in Group A relative to Group B. Of the 573 final differential features, 566 were annotated, while 7 remained unannotated (Figure 5b). The annotated features spanned flavonoids/isoflavonoids (89 features; 61 elevated in Group A), fatty acids/oxylipins (83; 38 elevated in Group B), terpenoids (9), phenolic derivatives (17), ellagitannins (4) and coumarins (1), together with other compound classes (363). (Figure 5c). The distribution of annotated compound classes indicates substantial chemical differences between the two groups.

Heatmap visualization of the top annotated discriminant features showed consistent abundance patterns across the biological replicates of each cultivar within each group (Figure 5d). Among the highest-VIP features elevated in Group A was a putatively annotated quercetin 4′-glucoside (positive-ion mode; Figure 5d). The guava-characteristic flavonoid glycoside avicularin was also enriched in Group A cultivars (‘Xigua’, ‘Hongbaoshi’, ‘Fenhongmi’), whereas isoquercitrin showed the opposite trend and was relatively more abundant in Group B; for these three flavonoid glycosides, the direction of difference was consistent across every cultivar of the two groups. Features preferentially elevated in Group B included several unsaturated fatty acid derivatives and aldehyde-class lipid oxidation products [51]. Taken together, these results indicate that the metabolic divergence between Group A and Group B is primarily characterized by differences in flavonoid glycosides and other phenolic secondary metabolites on the one hand, and lipid-derived compounds on the other.

### 3.5. Targeted Quantification of Flavonoids and Phenolic Compounds

A total of 21 compounds were targeted for quantification across eight guava tea cultivars, of which 15 were detected in at least one cultivar (Table 3 and Table S2). Five compounds—chlorogenic acid, cinnamic acid, rosmarinic acid, salvianolic acid A, and salvianolic acid B—were not detected in any sample and were excluded from further analysis. Myricetin was not detected in ‘Jindou Xiang’, while caffeine was absent in ‘Fenhongmi’, ‘Wuzi’, and ‘Xinshiji’ but present at comparable levels (≈72–75 μg kg^−1^ DW) in the remaining five cultivars.

Among detected compounds, quinic acid was the most abundant, ranging from 51,933 μg kg^−1^ DW in ‘Xinshiji’ to 83,807 μg kg^−1^ DW in ‘Fenhongmi’ (*p* < 0.05). Quercetin was the second-most abundant compound overall, with ‘Jindou Xiang’ recording the highest concentration (3588 μg kg^−1^ DW) and ‘Yanzhi Hong’ the lowest (1293 μg kg^−1^ DW). Vitexin and dihydromyricetin showed the greatest cultivar-dependent variation: vitexin ranged from 223 μg kg^−1^ DW (‘Wuzi’) to 1667 μg kg^−1^ DW (‘Jindou Xiang’), while dihydromyricetin was highest in ‘Hongbaoshi’ (1040 μg kg^−1^ DW) and lowest in ‘Yanzhi Hong’ (168 μg kg^−1^ DW). Notably, ‘Yanzhi Hong’ exhibited distinctively elevated phenolic acid content (423 μg kg^−1^ DW), approximately 1.6–2.1-fold higher than the other cultivars (*p* < 0.05). By contrast, coumalic acid, hesperidin, myricitrin, rutin, tangeretin, and protamine sulfate showed no significant differences among cultivars (*p* > 0.05).

### 3.6. Cultivar-Dependent Variation in Physicochemical Quality Indices of Guava Tea

**Figure 6:**
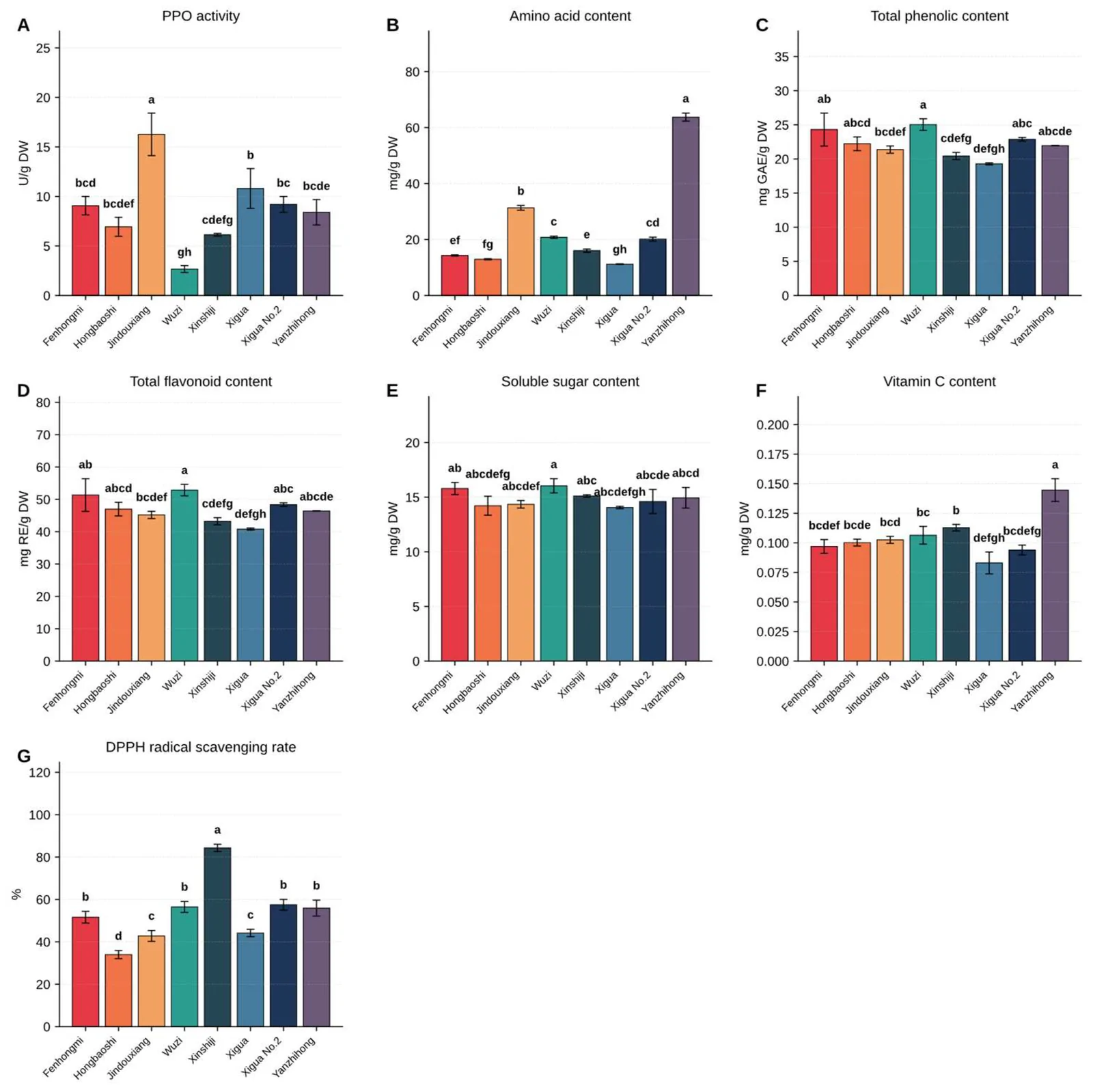
Physicochemical quality indices of eight guava tea cultivars and DPPH radical-scavenging capacity. (**A**) Polyphenol oxidase (PPO) activity, (**B**) amino acid content, (**C**) total phenolic content (TPC), (**D**) total flavonoid content (TFC), (**E**) soluble sugar content, and (**F**) vitamin C content were determined in eight guava cultivars. Data are presented as means ± standard error (SE) of three biological replicates. Different lowercase letters above bars indicate statistically significant differences according to Duncan’s multiple range test (*p* < 0.05). Cultivars are ordered alphabetically: Fenhongmi, Hongbaoshi, Jindou Xiang, Wuzi, Xinshiji, Xigua, Xigua No. 2, and Yanzhi Hong. DW, dry weight; GAE, gallic acid equivalents; RE, rutin equivalents. (**G**) DPPH radical-scavenging capacity.

Seven physicochemical indices—PPO activity, amino acid content, total phenolic content (TPC), total flavonoid content (TFC), soluble sugar content, vitamin C content and DPPH radical-scavenging capacity—were determined across the eight guava tea cultivars (Figure 6). All indices showed statistically significant cultivar-dependent variation, with each pairwise comparison fully resolved by Duncan’s multiple range test (*p* < 0.05), though the magnitude of variation differed considerably among traits.

PPO activity exhibited the widest relative variation among all indices measured, spanning a 6.1-fold range from 2.67 U/g DW in ‘Wuzi’ to 16.27 U/g DW in ‘Jindou Xiang’ (Figure 6A). The remaining cultivars were evenly distributed across eight distinct significance groups, with ‘Xigua’ (10.80 U/g DW) ranking second and ‘Xinshiji’ (6.13 U/g DW) second lowest. Amino acid content was similarly divergent across cultivars (Figure 6B). ‘Yanzhi Hong’ recorded by far the highest amino acid level (63.74 mg/g DW), exceeding the second-ranked ‘Jindou Xiang’ (31.32 mg/g DW) by more than two-fold and surpassing the lowest-ranked ‘Xigua’ (11.20 mg/g DW) by nearly six-fold. The remaining six cultivars clustered within a comparatively narrow range of 12.94–20.78 mg/g DW.

TPC and TFC displayed highly concordant cultivar rankings (Figure 6C,D): the order ‘Wuzi’ > ‘Fenhongmi’ > ‘Xigua No. 2′ > ‘Hongbaoshi’ > ‘Yanzhi Hong’ > ‘Jindou Xiang’ > ‘Xinshiji’ > ‘Xigua’ was identical for both indices, suggesting that flavonoids constitute a dominant and proportionally consistent fraction of the total phenolic pool across these cultivars. Absolute variation was more modest than for PPO or amino acids: TPC ranged from 19.29 (‘Xigua’) to 25.03 mg GAE/g DW (‘Wuzi’), a 1.3-fold difference, and TFC from 40.82 (‘Xigua’) to 52.87 mg RE/g DW (‘Wuzi’), likewise 1.3-fold.

Soluble sugar content showed the least relative variation of all six indices, ranging only 1.14-fold from 14.05 (‘Xigua’) to 16.04 mg/g DW (‘Wuzi’), yet all eight cultivars remained statistically distinguishable (Figure 6E). Vitamin C content ranged from 0.083 (‘Xigua’) to 0.145 mg/g DW (‘Yanzhi Hong’), a 1.74-fold difference (Figure 6F). ‘Yanzhi Hong’ was significantly higher than all other cultivars in vitamin C, while also leading in amino acid content, indicating that this cultivar possesses a distinct nutritional profile relative to the others. Conversely, ‘Wuzi’ ranked first in TPC, TFC, and soluble sugar content while recording the lowest PPO activity, DPPH radical-scavenging activity also differed significantly among cultivars (Figure 6G). ‘Xinshiji’ showed the highest activity, whereas ‘Hongbaoshi’ exhibited the lowest. ‘Wuzi, Xigua No. 2, Yanzhihong, and Fenhongmi’ showed intermediate activity, while ‘Jindouxiang and Xigua’ showed comparatively lower values. Notably, the cultivar ranking for DPPH activity did not parallel the rankings for TPC or TFC’, a combination that may reflect a divergent candidate model among these cultivars.

### 3.7. Integrated Candidate Analysis Prioritizes Quality-Associated Metabolite Subsets in Guava Leaf Tea

**Figure 7:**
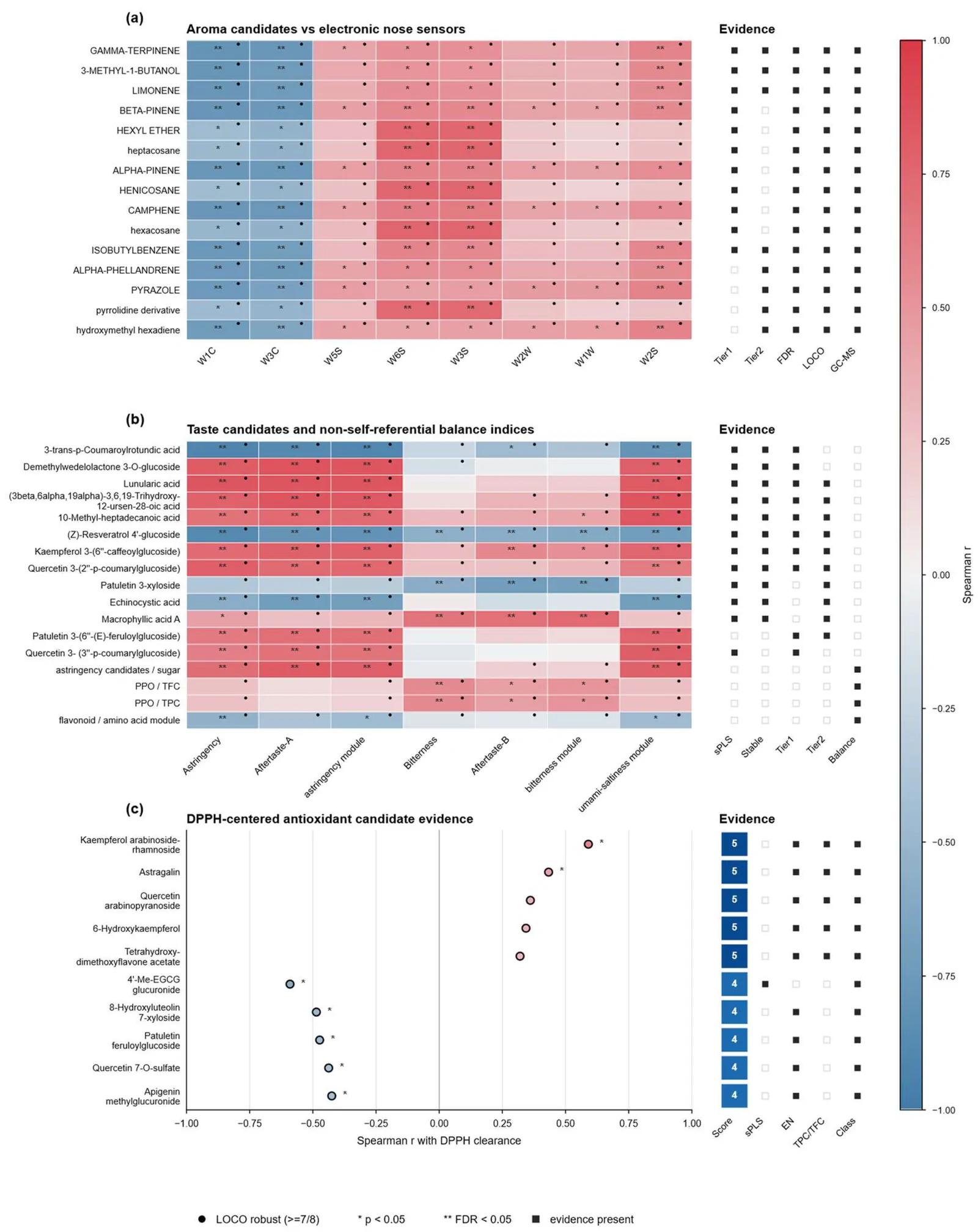
Integrated candidate analysis links quality-associated metabolite subsets with sensory and antioxidant-related traits in guava leaf tea. (**a**) Heatmap of aroma-related candidate volatile metabolites detected by GC–MS versus electronic nose sensors, with an adjacent evidence strip indicating reliability tier, FDR support, LOCO robustness, and confirmed GC–MS annotation. Panel a was not based on an sPLS model; aroma candidates were prioritized by correlation strength, FDR support, LOCO robustness, and annotation reliability. (**b**) Heatmap of untargeted UHPLC–QTOF-MS taste-related candidate non-volatile metabolites and non-self-referential balance indices versus electronic-tongue traits and taste modules, with an adjacent evidence strip indicating sPLS selection, LOCO stability, reliability tier, and balance-index status. (**c**) DPPH-centered display of antioxidant-related multi-evidence candidates, with an adjacent evidence strip summarizing evidence score, sPLS/ElasticNet selection, TPC/TFC co-direction, and antioxidant-class support. TPC, TFC, vitamin C, and PPO were used as supporting evidence layers rather than parallel response traits in the main figure. orange circles indicate positive correlations (r > 0; higher abundance associated with greater DPPH scavenging), whereas blue circles indicate negative correlations (r < 0; higher abundance associated with lower scavenging).

To link metabolite variation with the aroma, taste, and antioxidant-related quality traits described above, we performed an integrated candidate analysis using 24 matched biological samples from 8 cultivars (8 cultivars × 3 biological replicates). Metabolite and quality-index data were converted to platform-appropriate standardized relative abundance/intensity values before integrated analysis. GC–MS volatile features and UHPLC–QTOF-MS non-volatile features were log1p-transformed and Z-score-normalized, whereas UHPLC–TQ-MS/MS (MRM)-targeted analytes and physicochemical indices were Z-score-standardized before integrated association or evidence-layer analyses. Associations were evaluated using Spearman correlation, and robustness was assessed by leave-one-cultivar-out (LOCO) analysis grouped by cultivar. Across the aroma, taste, and antioxidant domains, the initial correlation screening identified 2012 nominally significant association pairs (*p* < 0.05). Subsequent evaluation based on FDR support, effect size, LOCO robustness, annotation reliability, and biological interpretability prioritized 53 Tier 1 core candidates and 57 Tier 2 robust exploratory candidates. Tier assignment incorporated correlation strength (|ρ|), *p*/FDR support, LOCO robustness, consistency of direction at the *n* = 8 cultivar-mean level, annotation reliability, and biological interpretability. The full Tier 1 and Tier 2 candidate lists, annotation metadata, and Figure 7 evidence layers are provided in Table S3, and the distribution of candidate tiers across quality domains is summarized in Figure S1.

For aroma-related traits, the integrated figure focused on 15 GC–MS-confirmed volatile candidates and their associations with electronic nose sensors (Figure 7a). Because panel A was not based on an sPLS model, the adjacent evidence strip summarizes the reliability tier, FDR support, LOCO robustness, and confirmed GC–MS annotation used for candidate prioritization. This analysis should be interpreted as candidate links between volatile composition and electronic nose response patterns, rather than as one-to-one assignment of individual sensors to single volatile metabolites. The final main-text panel uses electronic nose sensor outputs only.

Taste-associated modeling indicated that astringency-related phenotypes were better represented by combinations of candidate metabolites than by a single metabolite (Figure 7b). The sPLS models showed strong LOCO performance for astringency (Q^2^ = 0.733, ρ = 0.874, cultivar-level permutation *p* ≤ 0.001), Aftertaste-A (Q^2^ = 0.900, ρ = 0.978, *p* ≤ 0.001), and the taste_astringency_module (Q^2^ = 0.842, ρ = 0.951, *p* ≤ 0.001). The taste_umami_saltiness_richness_module also retained moderate predictive structure (Q^2^ = 0.576, ρ = 0.814, *p* = 0.003), whereas Aftertaste-B (Q^2^ = 0.188, *p* = 0.051), the taste_bitterness_module (Q^2^ = 0.120, *p* = 0.067) and bitterness (Q^2^ = 0.038, *p* = 0.081) did not reach significance, showed weak LOCO performance, and were not emphasized as robust main-text models. The Figure 7b evidence strip marks sPLS-selected candidates, LOCO-stable selected variables, reliability tiers, and balance-index status. The full sPLS model performance, selected variables, and LOCO stability-selection results are provided in Figure S2 and Table S3.

The displayed non-volatile taste candidates included putatively annotated UHPLC–QTOF-MS phenolic glycosides, flavonoid derivatives, triterpenoid acids, and saponin-related candidates (Figure 7b). Representative features were putatively annotated as demethylwedelolactone 3-O-glucoside, kaempferol 3-(6″-caffeoylglucoside), lunularic acid, 3-trans-p-coumaroylrotundic acid, (3β,6α,19α) −3,6,19-trihydroxy-12-ursen-28-oic acid, echinocystic acid, and (Z)-resveratrol 4′-glucoside. ElasticNet results for taste were treated with caution because the LOCO Q^2^ values were negative across the tested taste responses.

Balance-index analysis provided an additional view of taste-related associations by testing metabolite or quality-index ratios that were not self-referential to the response variable (Figure 7b). Among 91 tested balance-trait associations, 28 reached *p* < 0.05 and 13 remained significant at FDR < 0.05. Four non-self-referential balance indices were retained for the main figure (Figure 7b): total_astringency_candidates/soluble_sugar, PPO/TFC, PPO/TPC, and flavonoid_module/amino_acid_module. The total_astringency_candidates/soluble_sugar index was stably and positively associated with Aftertaste-A (r = 0.809, FDR = 5.21 × 10^−5^, LOCO 8/8), the taste_astringency_module (r = 0.790, FDR = 9.80 × 10^−5^, LOCO 8/8), and astringency (r = 0.717, FDR = 0.0011, LOCO 8/8). These results indicate a balance association between the astringency candidate set and soluble sugar, rather than a causal interpretation. The DPPH efficiency index (per TPC), DPPH efficiency index (per TFC), and polyphenol efficiency index (per TFC) were excluded from the main figure and retained in materials because they are derived efficiency indices and not independent predictors (Figure S3 and Table S3).

For antioxidant-related traits, the integrated analysis supported a DPPH-centered multi-evidence candidate set rather than a single confirmatory conclusion (Figure 7c). Five high-confidence candidates were prioritized: astragalin, putatively annotated kaempferol 3-(3″-acetyl-α-L-arabinofuranoside)-7-rhamnoside, putatively annotated quercetin 3-β-L-arabinopyranoside, putatively annotated 6-hydroxykaempferol, and putatively annotated 5,7,2′5′-tetrahydroxy-3,4′-dimethoxyflavone 5′-acetate. Among bulk antioxidant-related indices, TPC and TFC showed only weak correlations with DPPH clearance (both r = 0.188), whereas vitamin C showed a positive trend (r = 0.387). Thus, DPPH clearance was more appropriately described as associated with a distributed candidate set than with a single metabolite or with total phenolics/flavonoids alone.

In Figure 7c, DPPH clearance is the core endpoint, whereas TPC/TFC co-direction, sPLS/ElasticNet selection, antioxidant-class support, and evidence score are shown as supporting evidence layers rather than as parallel response traits. Structurally interpretable high- and medium-confidence candidates were retained in the main figure, while structurally high-risk UHPLC–QTOF-MS entries such as edulisin IV, vitamin D-like long-name features, 5-HOME (2), and 8-oxo-octadecanoic acid were moved to materials. The complete antioxidant evidence ranking, including candidates retained only in materials because of higher structural-annotation risk, is provided in Figure S4 and Table S3.

Across the aroma, taste, and antioxidant domains, the multi-evidence integration in Figure 7 identified a prioritized subset of 110 candidate quality-associated metabolites (53 Tier 1 core candidates and 57 Tier 2 exploratory candidates).

## 4. Discussion

### 4.1. Volatile Chemotype Divergence Reflects Distinct Product-Accumulation Patterns Rather than a Single Aroma Gradient

The tripartite volatile classification identified in the Results reflects three distinct volatile composition patterns rather than a single “more-or-less aroma” axis. The Group B vs. Group C OPLS-DA model fitted the data closely (R^2^Y = 0.994) [52,53]; however, because Group B and Group C comprised only two and three cultivars, respectively, the exact cultivar-level permutation test could not attain a *p* value below 0.10 (observed *p* = 0.10), and LOCO Q^2^ was negative (−0.200). This model is therefore used to rank the features that differ between the studied cultivars of the two groups, rather than as statistical confirmation of the two chemotypes. Within this model, Group B’s signature was both compositionally and quantitatively concentrated: monoterpenes accounted for 15 of 47 group-enriched differential metabolites but 96.4% of their combined abundance, with alpha-pinene and limonene together contributing the large majority of this pool. Notably, alpha-pinene, limonene, and beta-pinene also occupied the top three VIP ranks among all 77 strict differential metabolites (VIP = 1.92, exceeding the 95th percentile of 1.89), indicating that the compounds contributing most to the separation between Group B and Group C were the same compounds dominating Group B’s chemical composition [54,55]—a convergence that strengthens confidence in monoterpenes as the primary compositional basis of this chemotype [17,54] (Table S4). At the cultivar level, however, this signature was not uniformly expressed within Group B: total abundance of the 16 monoterpene markers was highest in ‘Yanzhi Hong’ (approximately 1.8- to 3.7-fold that of the other cultivars), whereas ‘Jindou Xiang’ was intermediate and similar to ‘Xinshiji’ from Group A (Supplementary Table S5). The negative LOCO Q^2^ values are consistent with this structure, in which the monoterpene-dominant signature is most pronounced in ‘Yanzhi Hong’.

Group C exhibited a different structural pattern. Its 26 enriched metabolites spanned 11 chemical classes, nearly doubling the effective diversity of Group B (Shannon effective categories: 9.0 vs. 5.1), indicating a compositionally more heterogeneous chemotype (Table S4). However, this class-level diversity did not translate into a correspondingly even abundance distribution: a single sesquiterpene ester, cis-nerolidyl acetate, accounted for 48.4% of total abundance among Group C-enriched metabolites [54,56], followed by an alkane fraction (28.4%, driven primarily by a chromatographically resolved eicosane isomer eluting at 18.84 min) and a ketone fraction (8.1%, including methyl 4-methyl-3-cyclohexenyl ketone) (Table S4). This pattern—many chemical classes represented, but abundance concentrated in a small subset—contrasts with Group B, where both class diversity and abundance were concentrated in the same pathway (monoterpene biosynthesis), and suggests that Group C’s aroma-related metabolism is not organized around a single dominant biosynthetic route but instead reflects the combined output of several structurally unrelated pathways [53,57], most prominently sesquiterpene, alkane, and ketone-related metabolism.

Group A’s status as a distinct metabolotype rests on a different kind of evidence. Rather than being defined by the enrichment of any particular compound or class, Group A was characterized by a lower abundance of most discriminant volatiles, with the large majority of differential metabolites reaching their lowest abundance in this group across both the Group A vs. Group B (202 differential metabolites) and Group A vs. Group C (175 differential metabolites) comparisons. This asymmetry, with few compounds elevated and many compounds depleted [58], distinguishes the compositional pattern of Group A from Groups B and C, whose signatures are characterized by the enrichment of different volatile compound classes. However, this pattern cannot by itself distinguish reduced biosynthetic output from other mechanisms affecting final headspace abundance. Lower precursor availability, altered enzymatic formation or degradation, differential volatilization during tea processing, and cultivar-dependent matrix retention could all contribute to the lower abundance of these volatiles in Group A. Accordingly, Group A is more appropriately interpreted as a chemotype characterized by the lower abundance of most discriminant volatiles, although its total volatile abundance overlapped with that of Group C, without inferring reduced activity of specific volatile-generating pathways.

The electronic nose results provide an independent, sensor-level line of evidence consistent with these compositional differences. The ten sensor responses did not shift together in a single direction across cultivars: within-module correlations were consistently strong (W1C–W3C: r = 0.963; W1C–W5C: r = 0.936; W1W–W2W: r = 0.991; W6S–W3S: r = 0.987), whereas cross-module correlations were consistently negative (W1C–W2S: r = −0.959; W3C–W6S: r = −0.864) [53,59]. These patterns identified two opposing groups of cross-sensitive sensor responses and therefore represent differences in volatile fingerprints rather than direct evidence of specific chemical classes. ‘Yanzhi Hong’ and ‘Hongbaoshi’ occupied opposite extremes on both PC1 and LD2, mirroring the compound-level contrast between concentrated monoterpene dominance and distributed non-monoterpene composition [7,60,61]. This convergence between an untargeted sensor-array method and a compound-resolved chromatographic method strengthens confidence that the contrasting volatile profiles represent reproducible cultivar-associated compositional differences rather than a platform-specific artifact [60,62,63].

### 4.2. Electronic-Tongue Response Differentiation Reflects Module-Specific Metabolite Associations, with a Clear Compositional Basis for Astringency but an Unresolved Signal for Bitterness

Electronic-tongue profiling resolved the instrumental taste-related responses into at least three largely independent response modules rather than a single intensity axis: an astringency–aftertaste module (astringency–Aftertaste-A, r = 0.956), an umami–saltiness module (r = 0.929), and a bitterness–aftertaste module (bitterness–Aftertaste-B, r = 0.929) [18,19,64]. Consistent with this modular structure, the predictive strength of candidate metabolite associations varied substantially across modules: sPLS models for astringency, Aftertaste-A, and the composite astringency module showed strong and LOCO-robust performance (Q^2^ = 0.733–0.900), the umami–saltiness-richness module retained moderate predictive structure (Q^2^ = 0.576), whereas the bitterness axis showed negligible explanatory power (detailed below).

The astringency module was underpinned by convergent evidence across independent analytical platforms. The two cultivars with the highest electronic-tongue astringency scores, ‘Fenhongmi’ (9.67) and ‘Xigua’ (9.41), both belonged to LC–MS Group A, the non-targeted metabolomic cluster defined by PCA and characterized in the OPLS-DA comparison by higher levels of flavonoid glycosides such as avicularin and quercetin 3′-glucoside [12,65]. Because the LC–MS grouping was derived purely from untargeted chemical profiles with no reference to sensory data, its alignment with the astringency ranking constitutes independent cross-platform support, analogous to the aroma chemotype convergence between electronic nose and GC–MS described above. Targeted quantification data reinforced this pattern at the level of an individual compound: dihydromyricetin concentrations in the three Group A cultivars (‘Fenhongmi’: 453; ‘Hongbaoshi’: 1040; ‘Xigua’: 591 μg kg^−1^ DW) were consistently higher than in all four Group B cultivars (‘Wuzi’: 395; ‘Xinshiji’: 320; ‘Jindou Xiang’: 256; ‘Xigua No. 2′: 278 μg kg^−1^ DW). Notably, this compositional signature was not simply proportional to bulk phenolic content, as total phenolic and flavonoid contents showed little association with astringency-related traits. The total_astringency_candidates/soluble_sugar index was robustly associated with the electronic-tongue astringency response, Aftertaste-A, and the astringency module (r = 0.717–0.809, FDR < 0.05, LOCO 8/8), suggesting that the instrumental astringency-related response is associated with the relative balance between candidate phenolic compounds and background matrix components rather than with absolute phenolic abundance alone. Because human sensory perception was not directly evaluated, these associations should not be interpreted as direct evidence of perceived astringency or sugar-mediated sensory masking [66,67].

The umami–saltiness-richness module retained only moderate predictive structure (Q^2^ = 0.576). This result is only partly consistent with amino acid content, the compound class conventionally linked to umami intensity [21,22]: ‘Xigua’ recorded the highest electronic-tongue umami score (14.44) despite the lowest amino acid content (11.20 mg/g DW) among all cultivars, whereas ‘Yanzhi Hong’ showed the opposite pattern, with the highest amino acid content (63.74 mg/g DW) but a comparatively low umami score (11.60).

Astringency and umami responses were significantly correlated (r = 0.815); however, this relationship should not be interpreted as evidence of a shared chemical origin. It may reflect covariance among cultivar profiles and/or a partial cross-response of the electronic-tongue sensors. Accordingly, the chemical basis of the umami response remains unresolved and requires validation using targeted taste-active compounds and independent sensory measurements.

By contrast, the bitterness axis showed only a narrow phenotypic range despite reaching statistical significance. Bitterness varied 1.4-fold across cultivars (14.01–20.22) and had the second-lowest F-statistic among the eight electronic-tongue attributes (F = 61.36), markedly smaller than the variation observed for astringency (4.9-fold), saltiness (4.5-fold), or Aftertaste-B (3.9-fold). This comparatively subtle differentiation likely limits the statistical power available for metabolite association, consistent with the negligible predictive performance of the corresponding sPLS model (Q^2^ = 0.038, cultivar-level permutation *p* = 0.081), which was not retained as a robust candidate model [19,64,68]. Caffeine, a plausible contributor to bitter taste [51], showed a qualitative presence/absence pattern among cultivars (detected at comparable levels of 72–75 μg kg^−1^ DW in five of eight cultivars, undetected in ‘Fenhongmi’, ‘Wuzi’, and ‘Xinshiji’) that did not track with the bitterness ranking; ‘Fenhongmi’, in which caffeine was undetected, nonetheless ranked among the cultivars with the highest bitterness scores. Individually quantified flavonoid compounds provided similarly limited support, with none of several targeted candidates showing a significant association with bitterness across the electronic-tongue attributes tested. Methodological coverage may further constrain metabolite attribution in this domain: the annotated differential features were dominated by phenolic and flavonoid-derived metabolites, and bitter-active compound classes that are less efficiently ionized or less well-represented in spectral libraries may therefore be underrepresented among the candidate features evaluated here [12,69]. Taken together, cultivar-level differences in bitterness in this study appear comparatively subtle and are not resolved to a confident metabolite- or pathway-level basis with the present analytical design, in contrast to the clearer compositional basis identified for astringency.

### 4.3. Cultivar-Level DPPH Activity Is Not Adequately Explained by Bulk Phenolic Indices

Among the six physicochemical indices measured across the eight guava tea cultivars, PPO activity showed the widest relative variation (6.1-fold, from 2.67 U/g DW in ‘Wuzi’ to 16.27 U/g DW in ‘Jindou Xiang’), while TPC and TFC displayed concordant cultivar rankings with comparatively modest variation (1.3-fold for both, highest in ‘Wuzi’ and lowest in ‘Xigua’), and vitamin C varied 1.74-fold, peaking in ‘Yanzhi Hong’. These compositional differences establish a clear cultivar-dependent backdrop against which the determinants of antioxidant performance can be examined.

Despite substantial cultivar-level variation in bulk composition, DPPH radical-scavenging activity was only weakly correlated with TPC and TFC (r = 0.188 for each) and showed a positive but non-significant relationship with vitamin C (r = 0.387). The cultivar rankings also differed markedly: ‘Xinshiji’ exhibited the highest DPPH activity, whereas ‘Wuzi’ showed the highest TPC and TFC. Thus, within the cultivars examined here, bulk phenolic and flavonoid contents alone did not adequately explain the variation in DPPH activity. Within the conditions of the DPPH assay, these results indicate that radical-scavenging activity was not strongly predicted by the absolute abundance of total phenolics or flavonoids. A likely structural reason is that DPPH radical-scavenging activity in plant extracts is inherently multi-source: it can draw on phenolic acids, flavonoids, vitamin C, carotenoids, and other redox-active constituents spanning multiple compound classes, each with distinct dose–response relationships and reaction kinetics. TPC and TFC each capture only a subset of these contributors, and the relative contribution of each class can vary independently across cultivars. Under these conditions, no single bulk-composition index would be expected to produce a strong or consistent correlation with DPPH clearance, because the underlying signal is distributed across too many chemically heterogeneous sources to be captured by any one aggregate measure. However, the weak correlations may also partly reflect the assay itself, because DPPH responses depend on reaction kinetics, extraction conditions, and matrix effects that can differentially influence the measured activity of individual antioxidants. Moreover, DPPH represents only one chemical radical-scavenging endpoint and does not capture all mechanisms contributing to antioxidant capacity. Therefore, the present results should be interpreted specifically as DPPH radical-scavenging activity rather than as a comprehensive measure of overall antioxidant capacity. At the candidate-metabolite level, the integrated analysis prioritized five metabolites associated with DPPH activity, including astragalin and four putatively annotated flavonol derivatives. These associations provide candidate-level support for a possible multi-compound contribution but do not establish these metabolites as determinants of DPPH activity.

Of these five, only astragalin was independently supported by targeted quantitation, providing traceable concentration data across all eight cultivars; the remaining four candidates relied solely on non-targeted putative annotation and lack independent structural confirmation. Notably, even at this narrower, compound-resolved level, no single candidate could be established as a confirmed determinant of DPPH performance, reinforcing, rather than resolving, the distributed-candidate interpretation already indicated by the non-targeted evidence. As a glycoside of kaempferol, astragalin possesses a mono-hydroxylated B-ring rather than the ortho-dihydroxyl (catechol-type) structure associated with the strongest phenolic radical-scavenging activity, providing a structural rationale consistent with its detection as only a nominal, rather than dominant, antioxidant-associated candidate [2,8]. Complex antioxidant phenotypes are commonly reported to resist single-compound attribution in plant extracts more broadly, with total capacity often reflecting additive or synergistic contributions across multiple constituents rather than one dominant compound [3]. Kaempferol-type flavonoids such as astragalin are furthermore widely reported as common constituents across *P. guajava* leaf phytochemical profiles in general, raising the possibility that its contribution reflects a baseline, species-wide component rather than a cultivar-differentiating factor—in which case the four structurally distinctive but unconfirmed putative candidates may warrant particular attention in future cultivar-resolved validation [65,70].

PPO activity may additionally mark a processing-relevant axis of variation: ‘Wuzi’ combined the lowest PPO activity with the highest TPC and TFC content, a pattern potentially consistent with a phenolic-retention-type candidate model [71,72]. The mechanistic implications of this combination are addressed together with the broader pathway-level evidence in the integrated model below.

Taken together, cultivar differences in DPPH activity were not strongly explained by TPC, TFC, vitamin C, or any single resolved candidate metabolite. A contribution from multiple compounds therefore remains a plausible candidate interpretation, but identifying the determinants of DPPH activity will require targeted quantification and independent validation.

### 4.4. Integrated Candidate Pathway-Output Framework for Cultivar-Dependent Quality Variation in Guava Leaf Tea

**Figure 8:**
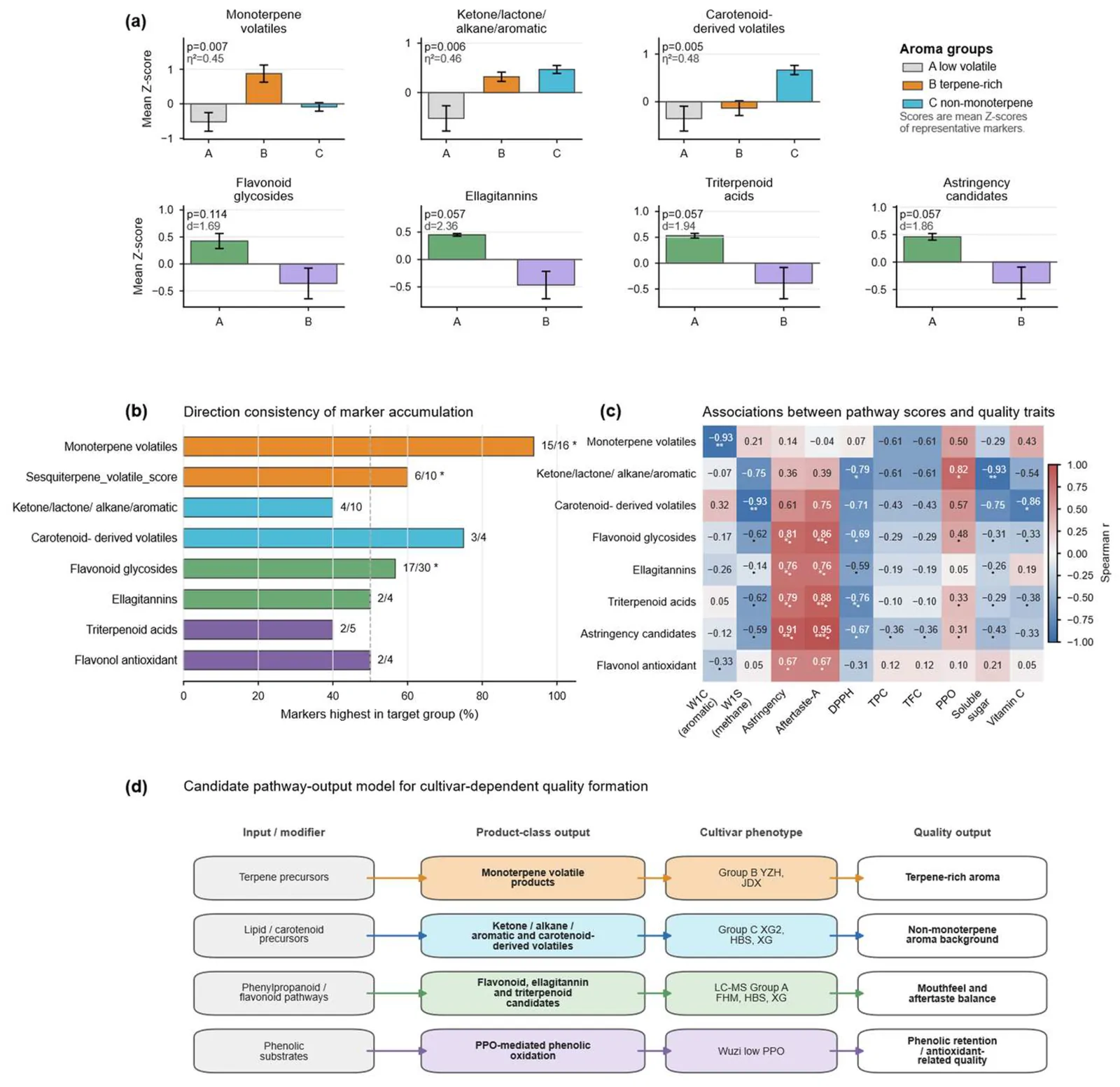
Candidate pathway-output framework for cultivar-dependent quality variation in guava leaf tea. (**a**) Pathway/product-class score comparison among aroma groups and LC–MS groups. Volatile pathway scores were compared among Aroma Groups A, B and C, whereas non-volatile pathway scores were compared between LC–MS Groups A and B. Scores were calculated as the mean Z-score of representative metabolites within each pathway/product class. Bars show group means with standard errors. Group differences are shown descriptively with effect sizes (η^2^ or Cohen’s d). (**b**) Direction consistency of representative pathway markers. Bars indicate the proportion of metabolites within each pathway/product class that reached their highest abundance in the hypothesized target group. Labels show enriched/total markers, and asterisks indicate binomial-test significance (* *p* < 0.05). (**c**) Correlation heat map between pathway/product-class scores, balance indices and quality traits. Cell color indicates Spearman correlation coefficients. Asterisks indicate statistical significance (* *p* < 0.05, ** *p* < 0.01, *** *p* < 0.001), and black dots indicate leave-one-cultivar-out stable associations (8/8), where applicable. (**d**) Schematic candidate pathway-output model linking upstream precursor or modifier layers, metabolite product classes, cultivar-associated phenotypes and quality outputs. The framework summarizes monoterpene-related aroma formation, non-monoterpene volatile formation, phenylpropanoid-/flavonoid-derived taste and antioxidant-related candidates, and PPO-mediated phenolic oxidation. The schematic represents candidate-level pathway-output evidence based on downstream product accumulation and does not indicate confirmed biosynthetic flux.

The multi-platform results were integrated into a candidate pathway-output framework describing associations between cultivar-dependent metabolite accumulation patterns and measured quality-related traits. To support this interpretation, representative pathway/product-class scores were calculated from downstream metabolite products and integrated with sensory and physicochemical quality traits (Figure 8). This analysis does not provide direct evidence of biosynthetic flux, but it provides quantitative support for cultivar-specific product-accumulation patterns that are relevant to aroma, taste, and antioxidant-related quality.

Several statistical limitations of the between-group analyses should be considered. Group labels were assigned to cultivars, so the cultivar rather than the individual replicate is the independent unit for assessing group differences. With only five to seven cultivars per comparison, exact permutation *p* values cannot fall below 0.10 (GC–MS) or 0.029 (LC–MS), and LOCO Q^2^ was negative for two of the three GC–MS comparisons and low for the positive-ion LC–MS model, indicating that some group separations depend on individual cultivars. The univariate between-group tests used to prioritize discriminant metabolites treated replicates as observations, so their *p* and FDR values serve as screening criteria rather than cultivar-level inference. In addition, the GC–MS aroma groups were defined by hierarchical clustering, and the LC–MS groups by PCA, of the same datasets used for OPLS-DA, which may enhance apparent separation. The group-level results are therefore interpreted descriptively for the eight studied cultivars, and the discriminant metabolites should be validated in independent cultivar sets before being generalized.

In addition to the statistical limitations, the structural evidence supporting the metabolites underlying the pathway/product-class scores should also be considered. Eight representative high-abundance metabolites were independently confirmed using authentic standards, whereas other candidate metabolites were supported by putative annotation based on accurate mass, isotope-pattern information, and/or MS/MS spectral matching. Detailed authentic-standard validation results are provided in Supplementary Table S6. Therefore, the pathway/product-class scores should be interpreted as candidate-level summaries of downstream metabolite accumulation rather than direct confirmation of pathway activity or biosynthetic flux.

The strongest pathway-output evidence was observed for the terpene-dominant aroma phenotype. Aroma Group B, consisting of ‘Yanzhi Hong’ and ‘Jindou Xiang’, showed a higher monoterpene-related volatile score than the other aroma groups, a difference driven mainly by ‘Yanzhi Hong’. More importantly, 15 of 16 monoterpene-related markers reached their highest abundance in this group, indicating that the terpene-dominant signature was not driven by a single compound but by the coordinated accumulation of multiple structurally related monoterpene products. These compounds included limonene, alpha-pinene, beta-pinene, camphene, alpha-terpinene, isoborneol, and monoterpene ester derivatives. Because these volatiles are downstream products of monoterpene metabolism [73,74,75], their coordinated enrichment supports a stronger monoterpene product-accumulation phenotype in Aroma Group B. This provides a plausible biochemical basis for the fresh, resinous, citrus-like, and herbal aroma background of these cultivars. Aroma Group C (‘Xigua No. 2′, ‘Hongbaoshi’, and ‘Xigua’) showed a different volatile-output pattern. This group had elevated ketone/lactone/alkane/aromatic volatile scores and higher carotenoid-derived volatile candidate scores, including ionol-related compounds. Compared with the terpene-dominant Group B, this pattern suggests a distinct non-monoterpene volatile background that may involve lipid-derived, wax-associated, processing-related, or carotenoid-derived volatile formation [76,77,78]. Therefore, aroma differentiation among the studied cultivars appears to involve two complementary composition patterns: monoterpene enrichment, most pronounced in ‘Yanzhi Hong’, and a non-monoterpene volatile background in sesquiterpene ester-/alkane-/ketone-associated cultivars. Group A cultivars were instead characterized by lower abundance of most discriminant volatiles rather than by the enrichment of a specific volatile subclass.

Pathway-score analysis for the integrated Figure 8 model corroborated this phenylpropanoid-/flavonoid-derived differentiation between LC–MS Group A and Group B at the product-class level, with Group A (‘Fenhongmi’, ‘Hongbaoshi’, ‘Xigua’) showing higher flavonoid glycoside, ellagitannin/hydrolyzable tannin, and triterpenoid acid/saponin-related scores than Group B. This pathway-score evidence should be treated as moderate rather than confirmatory, as it was calculated from cultivar-level data and many underlying UHPLC–QTOF-MS annotations were putative rather than confirmed by authentic standards.

The antioxidant-related results also argue against a simple total-phenolic model. DPPH clearance was weakly associated with total phenolic and total flavonoid contents [11,13,23], indicating that antioxidant performance among cultivars was not determined merely by bulk phenolic or flavonoid abundance. Although several flavonol-centered candidates, including astragalin, kaempferol derivatives, quercetin glycosides, and hydroxykaempferol-like compounds, were prioritized in the integrated analysis, the pathway-score confirmation did not provide strong evidence that a single flavonol score fully explained DPPH variation. Therefore, DPPH activity should be interpreted as a distributed functional phenotype that may depend on specific compound structures, oxidation state, extraction efficiency, matrix interactions, and additional unmeasured antioxidant contributors. The flavonol-specific antioxidant model remains exploratory and requires compound-level validation.

PPO activity may provide an additional processing-related layer linking phenolic composition with quality formation. ‘Wuzi’ showed a phenolic-retention-like phenotype characterized by relatively high total phenolic and flavonoid contents together with the lowest PPO activity [79,80,81]. This pattern is consistent with the possibility that lower PPO activity may reduce the oxidative conversion of phenolic substrates and favor phenolic retention during processing. In contrast, cultivars with higher PPO activity may have greater potential for phenolic oxidation, which could affect color development, astringency, aftertaste, and antioxidant performance. However, because oxidation products and processing time-course dynamics were not directly measured, the role of PPO should be interpreted as an inferential modifier rather than as a confirmed causal mechanism.

Taken together, these findings support a candidate pathway-output model for guava leaf tea quality formation. Aroma quality is mainly shaped by the balance between monoterpene accumulation and non-monoterpene volatile formation, taste quality is influenced by specific non-volatile candidate subsets and their balance with soluble sugars and amino acids, and antioxidant-related quality cannot be adequately predicted by total phenolic or flavonoid content alone. Importantly, the aroma grouping and the non-volatile LC–MS grouping did not completely overlap, suggesting that volatile aroma and non-volatile taste/functional traits are associated with partially independent metabolic axes. This has practical implications for cultivar selection: ‘Yanzhi Hong’, which showed the highest monoterpene abundance among the studied cultivars, may be the most promising candidate for developing terpene-rich aromatic products, with ‘Jindou Xiang’ showing intermediate monoterpene levels; ‘Xigua No. 2′, ‘Hongbaoshi’, and ‘Xigua’ are potentially suitable for providing a distinct non-monoterpene volatile background; ‘Fenhongmi’, ‘Hongbaoshi’, and ‘Xigua’ may potentially contribute stronger non-volatile phenolic/flavonoid-derived mouthfeel features; and ‘Wuzi’ may represent a candidate phenolic-retention phenotype.

Several limitations should be acknowledged. First, pathway/product-class scores were based on downstream metabolite abundance and therefore cannot prove increased biosynthetic flux. Second, many UHPLC–QTOF-MS-derived non-volatile candidates were putatively annotated and require confirmation using authentic standards. Third, LC–MS group comparisons were performed at the cultivar-mean level, which limits formal statistical power. Fourth, the distinction between statistical significance and biological meaningfulness remains interpretive: although fold-change thresholds and the multi-tier candidate framework were designed to prioritize metabolites with both strong statistical support and large effect sizes, the biological relevance of individual candidate metabolites must ultimately be confirmed by targeted quantification, purified-compound assays, or sensory recombination experiments. Finally, antioxidant and taste associations remain candidate-level evidence and should be validated by targeted quantification, purified-compound assays, recombination experiments, enzyme activity measurements, processing time-course analysis, and isotope tracing. Despite these limitations, the integrated pathway-output model provides a useful framework for connecting cultivar-specific metabolic composition with aroma, taste, and functional quality in guava leaf tea.

## 5. Conclusions

This multi-platform, cultivar-resolved study of guava leaf tea across eight cultivars shows that quality differences arise from coordinated, multi-trait phenotypes rather than isolated markers. Among the studied cultivars, volatile profiles were described by three composition patterns: monoterpene-concentrated (most pronounced in ‘Yanzhi Hong’), compositionally diverse non-monoterpene, and lower abundance of most discriminant volatiles, consistent across electronic nose and GC–MS data. The electronic-tongue astringency response showed a clear association with flavonoid-glycoside-enriched cultivars, whereas the instrumental bitterness response remained statistically detectable but compositionally unresolved. DPPH radical-scavenging activity was not strongly explained by bulk phenolic or flavonoid content, while the integrated analysis identified several candidate metabolite associations requiring further validation.

Integrating these layers into a candidate pathway-output framework suggests that aroma, instrumental taste-related responses, and antioxidant-related quality are associated with partially independent metabolic composition axes, offering practical guidance for cultivar-oriented product development toward terpene-rich, non-monoterpene, or phenolic-retention-type profiles. These conclusions remain candidate-level: pathway scores reflect downstream abundance rather than confirmed flux, and taste- and antioxidant-related associations require validation through targeted quantification and processing time-course analysis before the model can be considered mechanistically confirmed.

## Figures and Tables

**Table 1:** Electronic nose sensor responses and one-way ANOVA results for eight guava cultivars.

Sensor	XG	XG2	YZH	JDX	XSJ	HBS	WZ	FHM	F	*p*
W1C	0.74 e	0.76 b	0.63 h	0.73 f	0.73 g	0.81 a	0.75 c	0.75 d	26.1 ***	<0.001
W5S	16.3 b	11.9 e	18.4 a	12.8 c	12.8 c	6.87 h	10.8 f	8.81 g	5.5 **	0.002
W3C	0.94 g	0.95 d	0.90 h	0.94 f	0.95 e	0.96 a	0.95 b	0.95 c	24.0 ***	<0.001
W6S	1.01 b	1.01 e	1.03 a	1.01 c	1.00 f	1.01 d	1.00 h	1.00 g	16.5 ***	<0.001
W5C	0.58 e	0.58 d	0.49 h	0.58 c	0.56 g	0.66 a	0.57 f	0.59 b	15.3 ***	<0.001
W1S	1.92 h	2.30 d	2.55 a	2.12 f	2.30 c	1.93 g	2.32 b	2.26 e	6.4 **	0.001
W1W	22.2 b	20.5 e	25.8 a	20.7 d	20.7 c	14.0 h	20.3 f	17.1 g	6.4 **	0.001
W2S	1.22 f	1.22 g	1.42 a	1.24 e	1.27 b	1.17 h	1.25 c	1.24 d	13.7 ***	<0.001
W2W	17.5 b	15.4 d	19.6 a	15.6 c	15.3 e	10.8 h	15.1 f	12.8 g	5.8 **	0.002
W3S	1.03 b	1.01 e	1.04 a	1.02 c	1.01 f	1.01 d	1.00 h	1.00 g	21.7 ***	<0.001

Note. YZH = Yanzhi Hong, FHM = Fenhongmi, JDX = Jindou Xiang, XSJ = Xinshiji, HBS = Hongbaoshi, XG2 = Xigua No. 2, WZ = Wuzi, XG = Xigua. Sensor descriptions: W1C (aromatic compounds, benzene-like), W5S (nitrogen oxides), W3C (ammonia, aromatic compounds), W6S (hydrogen), W5C (alkanes, aromatic compounds, small polar molecules), W1S (methane), W1W (sulfides, terpenes), W2S (alcohols, aldehydes and ketones), W2W (aromatic compounds, organic sulfides), W3S (long-chain alkanes). Because MOS sensors are cross-sensitive, these categories indicate relative sensitivity rather than compound-class specificity. Values are means with Duncan grouping letters (*n* = 3). Different letters indicate significant differences (*p* < 0.05). ** *p* < 0.01, *** *p* < 0.001.

**Table 2:** Electronic-tongue taste attribute responses and one-way ANOVA results for eight guava tea cultivars.

Attribute	XG	XG2	YZH	JDX	XSJ	HBS	WZ	FHM	F	*p*
Aftertaste-B	4.75 ± 0.08 ef	3.34 ± 0.09 g	6.21 ± 0.37 bc	6.96 ± 0.05 a	5.30 ± 0.05 d	4.82 ± 0.01 e	1.78 ± 0.10 h	6.51 ± 0.03 b	435.8	4.82 × 10^−17^
Astringency	9.41 ± 0.20 ab	2.81 ± 0.07 g	6.00 ± 0.36 c	3.87 ± 0.04 ef	4.06 ± 0.17 e	5.82 ± 0.08 cd	1.98 ± 0.08 h	9.67 ± 0.26 a	704.85	1.05 × 10^−18^
Saltiness	5.14 ± 0.13 a	1.15 ± 0.09 gh	1.55 ± 0.07 f	2.38 ± 0.03 de	2.81 ± 0.03 c	2.48 ± 0.10 d	1.18 ± 0.11 g	4.66 ± 0.05 b	995.42	6.70 × 10^−20^
Aftertaste-A	4.87 ± 0.07 b	2.75 ± 0.05 h	3.88 ± 0.31 d	3.38 ± 0.02 ef	3.53 ± 0.04 e	4.50 ± 0.07 c	3.00 ± 0.07 g	5.19 ± 0.07 a	159.5	1.37 × 10^−13^
Bitterness	18.20 ± 0.11 e	17.51 ± 0.14 f	19.55 ± 1.21 ab	20.22 ± 0.05 a	19.62 ± 0.02 c	17.05 ± 0.15 g	14.01 ± 0.33 h	19.57 ± 0.03 cd	61.36	2.26 × 10^−10^
Sourness	−32.91 ± 0.06 h	−32.13 ± 0.07 g	−29.62 ± 0.88 ab	−31.42 ± 0.03 d	−31.95 ± 0.06 ef	−30.28 ± 0.13 a	−30.97 ± 0.05 c	−31.84 ± 0.04 e	33.91	1.94 × 10^−08^
Umami	14.44 ± 0.11 a	12.00 ± 0.03 d	11.60 ± 0.58 ef	11.90 ± 0.06 de	12.70 ± 0.09 c	11.57 ± 0.12 fg	11.07 ± 0.15 h	13.70 ± 0.04 b	79.78	3.02 × 10^−11^
Richness	2.60 ± 0.03 c	1.72 ± 0.07 fg	2.57 ± 0.08 cd	2.09 ± 0.06 e	1.79 ± 0.03 f	2.72 ± 0.06 ab	1.52 ± 0.01 h	2.80 ± 0.02 a	302.94	8.64 × 10^−16^

Note. YZH = Yanzhihong, FHM = Fenhongmi, JDX = Jindouxiang, XSJ = Xinshiji, HBS = Hongbaoshi, XG = Xigua, XG2 = Xigua No. 2, WZ = Wuzi. Taste attributes: Aftertaste-B (bitter aftertaste), Aftertaste-A (astringent aftertaste). Values are means with Duncan grouping letters (*n* = 3). Different letters indicate significant differences (*p* < 0.05).

**Table 3:** Contents of nine compounds showing significant differences among eight guava tea cultivars (μg kg^−1^ DW).

Compound	XG	XG2	YZH	JDX	XSJ	HBS	WZ	FHM
Quinic Acid	54,151 ± 2216 c	70,983 ± 1282 b	71,092 ± 6987 b	70,620 ± 1803 b	51,933 ± 6826 c	78,285 ± 4501 ab	73,959 ± 4508 b	83,807 ± 6738 a
Quercetin	3144 ± 87 b	3152 ± 166 b	1293 ± 208 d	3588 ± 211 a	3402 ± 224 ab	2565 ± 77 c	3084 ± 214 b	2329 ± 141 c
Vitexin	281 ± 8 d	907 ± 27 c	988 ± 49 b	1667 ± 43 a	965 ± 44 b	249 ± 25 d	223 ± 6 d	224 ± 25 d
Dihydromyricetin	591 ± 98 b	278 ± 13 de	168 ± 17 e	256 ± 13 de	320 ± 21 cd	1040 ± 175 a	395 ± 28 cd	453 ± 57 c
Myricetin	1463 ± 2 a	1463 ± 0 a	1460 ± 6 a	0 ± 0 b	1463 ± 1 a	1463 ± 0 a	1464 ± 0 a	1459 ± 8 a
Phenolic Acid	236 ± 31 bcd	201 ± 18 d	423 ± 22 a	219 ± 23 cd	246 ± 4 bc	258 ± 35 bc	267 ± 12 b	225 ± 1 cd
Astragalin	160 ± 2 bc	166 ± 2 a	160 ± 4b c	163 ± 3 ab	166 ± 3 a	156 ± 3 c	157 ± 4 c	162 ± 2 abc
Caffeic Acid	138 ± 3 b	140 ± 1 b	144 ± 3 a	139 ± 0 b	139 ± 2 b	138 ± 1 b	138 ± 1 b	138 ± 1 b
Caffeine	75 ± 3 a	75 ± 4 a	73 ± 1 a	72 ± 1 a	0 ± 0 b	74 ± 2 a	0 ± 0 b	0 ± 0 b

Note: Values are mean ± standard deviation. Different lowercase letters indicate significant differences among cultivars (Duncan’s multiple range test, *p* < 0.05). FHM = Fenhongmi, HBS = Hongbaoshi, JDX = Jindou Xiang, WZ = Wuzi, XG = Xigua, XG2 = XiguaNo2, XSJ = Xinshiji, YZH = Yanzhi Hong.

## Data Availability

The original contributions presented in this study are included in the article/Supplementary Material. Further inquiries can be directed to the corresponding authors.

## Supplementary Materials

The following supporting information can be downloaded at: https://www.mdpi.com/article/10.3390/foods15203616/s1, Table S1: OPLS-DA model fit and cultivar-level validation for pairwise comparisons of guava cultivars; Table S2: Contents of six compounds showing no significant variation among eight guava tea cultivars (μg kg^−1^ DW); Table S3: Full Tier 1 and Tier 2 candidate lists with annotation metadata; Table S4: OPLS-DA-derived differential volatile metabolites between Group B and Group C aroma metabolotypes of guava leaf tea; Table S5: Monoterpene and total volatile abundance of the eight cultivars; Table S6: Structural-confidence summary of metabolites underlying the pathway/product-class scores used in the integrated analysis; Figure S1: Candidate reliability-tier distribution across quality domains; Figure S2: Taste sPLS model performance under leave-one-cultivar-out (LOCO) validation.; Figure S3: Efficiency-index heatmap retained for supplementary interpretation; Figure S4: Antioxidant evidence landscape for DPPH-centered candidate prioritization; Figure S5-1: Authentic-standard confirmation of representative monoterpenes and oxygenated monoterpenes detected by GC–MS; Figure S5-2: Confirmation of selected metabolites by authentic reference standards using LC–QTOF–MS.
