# Supplementary material for: Multi-Platform Metabolomic and Sensory Profiling Reveals Cultivar-Dependent Volatile, Taste, and Antioxidant Chemotypes in Guava Leaf Tea
Source: Foods. 2026 Oct 11;15(20):3616. doi: 10.3390/foods15203616 (PMC13704376; doi:10.3390/foods15203616)
Supplement: Supplementary file 1 [file foods-15-03616-s001.zip › Supplementary_Materials_revised.pdf]

Supplementary Materials

Table S1. OPLS-DA model fit and cultivar-level validation for pairwise comparisons of guava cultivars.

Platform	Comparison	Cultivars	Samples	Assignments	R^2Y	LOCO Q^2	p (Q^2)	p (R^2Y)	Min. p
GC–MS	A vs B	3 vs 2	9 vs 6	10	0.998	−0.307	0.20	0.10	0.10
GC–MS	A vs C	3 vs 3	9 vs 9	20[a]	0.988	0.317	0.10	0.30	0.10
GC–MS	B vs C	2 vs 3	6 vs 9	10	0.994	−0.200	0.10	0.20	0.10
LC–MS (negative ion)	A vs B	3 vs 4	9 vs 12	35	0.997	0.414	0.029	0.029	0.029
LC–MS (positive ion)	A vs B	3 vs 4	9 vs 12	35	0.997	0.091	0.029	0.029	0.029

Note: GC–MS aroma groups: A = 'Wuzi', 'Fenhongmi', 'Xinshiji'; B = 'Yanzhi Hong', 'Jindou Xiang'; C = 'Xigua No. 2', 'Hongbaoshi', 'Xigua'. LC–MS groups: A = 'Fenhongmi', 'Hongbaoshi', 'Xigua'; B = 'Wuzi', 'Xinshiji', 'Jindou Xiang', 'Xigua No. 2'; 'Yanzhi Hong' was not assigned. Biological replicates were used as observational rows and cultivars as the unit of validation and permutation. R^2Y refers to the full-data fit. LOCO Q^2, leave-one-cultivar-out Q^2 (all replicates of a cultivar held out together), used as a diagnostic of the stability of group separation. Assignments, number of distinguishable assignments of group labels to cultivars; p (Q^2) and p (R^2Y), exact cultivar-level permutation p values obtained by refitting and revalidating the model under every assignment; Min. p, smallest attainable p value for the design. [a] The 20 assignments form 10 mirror-image pairs with identical statistics, so the smallest attainable p value is 0.10.

Table S2. Contents of six compounds showing no significant variation among eight guava tea cultivars (μg kg^{-1} DW).

Compound	XG	XG2	YZH	JDX	XSJ	HBS	WZ	FHM
Coumalic acid	891±48	923±5	917±6	915±14	918±11	921±4	915±14	920±8
Hesperidin	111±6	107±2	112±2	106±4	110±4	107±3	111±7	108±3
Myricitrin	160±1	160±1	156±7	161±1	160±1	162±3	160±2	160±0
Rutin	111±7	107±2	112±2	106±4	110±4	107±3	110±8	108±3
Protamine Sulfate	96±4	94±2	95±2	94±0	96±4	94±3	96±5	93±1
Tangeretin	247±4	245±0	245±1	246±2	245±0	245±0	245±0	245±0

Note: Values are mean ± standard deviation. ns indicates no significant difference among cultivars (one-way ANOVA, $p > 0.05$).

Table S4-1. OPLS-DA-derived differential volatile metabolites (VIP > 1, FDR < 0.05) between Group B and Group C aroma metabolotypes of guava leaf tea.

Metabolite	Compound class	VIP	Fold change (B/C)	log2FC	P value	FDR	Higher in	Mean Group B	Mean Group C	p (correlation)	Total score	Fill %	Note
ALPHA-PINENE_ID123	Monoterpene (incl. esters/alcohols/ketones)	1.923	72.43	6.18	0.000001	0.00009	B	110344509.7	1523498.3	0.979			
LIMONENE	Monoterpene (incl. esters/alcohols/ketones)	1.922	31.76	4.99	0	0	B	43703009.5	1375844	0.991			
BETA-PINENE_ID150	Monoterpene (incl. esters/alcohols/ketones)	1.917	16.27	4.02	0.000001	0.00009	B	2688712.3	165260.4	0.965			
Unknown_ID120	Other	1.892	61.2	5.94	0	0.000006	B	448284.5	7324.6	0.949			
2-ISOPROPYL-3-METHYLPYRAZINE	Pyrazine	1.891	14.5	3.86	0	0	B	64910.8	4478.1	0.972			
ISOBORNEOL_ID296	Monoterpene (incl. esters/alcohols/ketones)	1.889	14.54	3.86	0.000162	0.004333	B	274312.7	18871.3	0.923			
MYRTENYL ACETATE_ID277	Monoterpene (incl. esters/alcohols/ketones)	1.888	15.16	3.92	0	0	B	56115.2	3702.1	0.973			
BETA-PINENE_ID161	Monoterpene (incl. esters/alcohols/ketones)	1.888	4.46	2.16	0.00038	0.007873	B	2815727.5	631044.7	0.887			
(E,E)-farnesol_ID162	Sesquiterpene (incl. esters/alcohols)	1.887	4.16	2.06	0.000426	0.008398	B	1777069.5	427595.6	0.88			
ALPHA-FENCHYL ACETATE	Monoterpene (incl. esters/alcohols/ketones)	1.876	7.1	2.83	0	0.000013	B	136412.8	19205.9	0.942			
ALPHA-PHELLANDRENE_ID168	Monoterpene (incl. esters/alcohols/ketones)	1.875	5.26	2.4	0.001583	0.020812	B	348953	66312.9	0.788			
ALPHA-TERPINENE_ID122	Monoterpene (incl. esters/alcohols/ketones)	1.851	20.97	4.39	0.0001	0.002956	B	247137.8	11783.4	0.933			
Unknown_ID252	Other	1.846	10.13	3.34	0.000002	0.000135	B	38420	3792.4	0.918			
Unknown_ID130	Other	1.838	14.39	3.85	0.000073	0.002238	B	48500.8	3370.3	0.89			

**ISOBUTYLBENZENE_ID131**	**Aromatic**	**1.832**	**5.85**	**2.55**	**0.000012**	**0.000645**	**B**	**61416.7**	**10507.3**	**0.875**			
**Unknown_ID128**	**Other**	**1.831**	**9.13**	**3.19**	**0.00096**	**0.01445**	**B**	**28865.3**	**3162.9**	**0.834**			
**PYRAZOLE**	**Other**	**1.827**	**6.31**	**2.66**	**0.00001**	**0.000565**	**B**	**56629**	**8974.4**	**0.881**			
**CAMPHENE_ID129**	**Monoterpene (incl. esters/alcohols/ketones)**	**1.814**	**18.84**	**4.24**	**0.000041**	**0.001457**	**B**	**236732.5**	**12565.4**	**0.883**			
**Unknown_ID395**	**Other**	**1.8**	**6.68**	**2.74**	**0.000319**	**0.007146**	**B**	**9007.8**	**1348.7**	**0.827**			
**ISOBUTYLBENZENE_ID133**	**Aromatic**	**1.797**	**5.07**	**2.34**	**0.000035**	**0.00131**	**B**	**61416.7**	**12122.8**	**0.849**			
**Unknown_ID275**	**Other**	**1.783**	**4.47**	**2.16**	**0.000016**	**0.000754**	**B**	**28791**	**6439.4**	**0.872**			
**Unknown_ID73**	**Other**	**1.78**	**4.44**	**2.15**	**0.003659**	**0.038345**	**B**	**124682.5**	**28076.7**	**0.774**			
**BORNYL FORMATE**	**Monoterpene (incl. esters/alcohols/ketones)**	**1.779**	**5.08**	**2.35**	**0**	**0.000064**	**B**	**84875.5**	**16697.3**	**0.918**			
**BORNYL PROPIONATE**	**Monoterpene (incl. esters/alcohols/ketones)**	**1.774**	**4.95**	**2.31**	**0.000001**	**0.000068**	**B**	**85165.5**	**17218.1**	**0.916**			
**DIMETHYL TRISULFIDE**	**Sulfur compound**	**1.765**	**8.06**	**3.01**	**0.000534**	**0.010053**	**B**	**39848.3**	**4946.2**	**0.806**			
**3-HYDROXY-4-HYDROXYMETHYL-3,5-DIMETHYL-1,5-HEXADIENE**	**Alcohol/other**	**1.755**	**6.47**	**2.69**	**0.000013**	**0.000645**	**B**	**56491.7**	**8732.8**	**0.864**			
**neryl acetate**	**Monoterpene (incl. esters/alcohols/ketones)**	**1.755**	**4.32**	**2.11**	**0.001542**	**0.020596**	**B**	**43681.5**	**10121.6**	**0.845**			
**Y-METHYLBICYCLO(4.3.0)NON-6-EN-8-ONE**	**Other**	**1.748**	**5.92**	**2.57**	**0.000007**	**0.000452**	**B**	**16560.7**	**2797.8**	**0.869**			
**Oxybuprocaine**	**Other**	**1.734**	**3.27**	**1.71**	**0.00065**	**0.011479**	**B**	**39666**	**12140.2**	**0.836**	0.739	**8**	**Low-confidence library match (Total score < 0.80) with low detection rate; chemically implausible as a tea-derived compound (local anesthetic). Excluded from interpretation.**

DIMETHYL THIOETHER	Sulfur compound	1.717	5.47	2.45	0.000336	0.007314	B	1696069.3	310106.3	0.829	
METHYL VINYL KETONE_ID12	Ketone	1.708	2.27	1.18	0.00005	0.001588	B	562877.7	248353.1	0.855	
BUTANE	Alkane	1.708	2.27	1.18	0.00005	0.001588	B	562877.7	248353.1	0.855	
Unknown_ID281	Other	1.702	5.19	2.38	0.000012	0.000645	B	16349	3151.2	0.873	
Unknown_ID305	Other	1.698	3.24	1.7	0.000026	0.001038	B	15380.5	4739.9	0.837	
Unknown_ID49	Other	1.677	4.02	2.01	0.003074	0.033932	B	6466	1607.9	0.75	
Unknown_ID278	Other	1.676	4.78	2.26	0.000021	0.000897	B	40790.2	8528.3	0.851	
Unknown_ID292	Other	1.631	4.64	2.21	0.000859	0.013681	B	17221	3709.2	0.766	
Unknown_ID135	Other	1.625	5.5	2.46	0.000683	0.011544	B	15027.3	2729.9	0.843	
Unknown_ID374	Other	1.622	3.24	1.7	0.002217	0.0272	B	10265.2	3166.7	0.796	
Unknown_ID164	Other	1.616	2.62	1.39	0.004413	0.042961	B	56907.7	21750.2	0.72	
(E)-3,7-DIMETHYL-2,6-OCTADIEN-1-OL	Other	1.615	3.2	1.68	0.000123	0.00351	B	72040.8	22545.7	0.801	
Unknown_ID279	Other	1.61	5.13	2.36	0.000042	0.001457	B	16349.3	3188.9	0.834	
hexacosane_ID473	Alkane	1.598	3.12	1.64	0.000171	0.004432	B	72091.5	23096.2	0.791	
(1S-TRANS)-2-METHYL-5-(1-METHYLETHENYL)-2-CYCLOHEXEN-1-OL_ID443	Other	1.592	2.65	1.41	0.00071	0.011757	B	15318.2	5781.1	0.775	
Unknown_ID69	Other	1.549	4.87	2.28	0.002234	0.0272	B	54182.5	11129.8	0.822	
Unknown_ID125	Other	1.542	5.48	2.45	0.00285	0.032327	B	33760.3	6161.8	0.672	
(R)-2-METHYL-5-(1-METHYLETHENYL)-2-CYCLOHEXEN-1-ONE	Ketone	1.507	3.41	1.77	0.000638	0.011479	B	17692.3	5193.1	0.765	
Unknown_ID124	Other	1.457	6.48	2.7	0.003528	0.037456	B	55200.5	8517.1	0.733	
sesquisabinene	Sesquiterpene (incl. esters/alcohols)	1.889	0.2	-2.33	0.003354	0.036071	C	2301128.5	11580527.7	-0.823	Same peak as CIS-NEROLIDYL ACETATE (RT diff

											0.002 min); count once		
CIS-NEROLIDYL ACETATE	Sesquiterpene (incl. esters/alcohols)	1.889	0.2	-2.33	0.003354	0.036071	C	2301128.5	11580527.7	-0.823	Same peak as sesquisabinene (RT diff 0.002 min); count once		
Unknown_ID267	Other	1.884	0.41	-1.29	0.002148	0.026952	C	301066	736918.4	-0.866			
BETA-BUTYROLACTONE	Lactone	1.881	0.41	-1.27	0.000406	0.008194	C	255765.2	617465.7	-0.853			
METHYL 4-METHYL-3-CYCLOHEXENYL KETONE	Ketone	1.88	0.35	-1.53	0.004462	0.042961	C	459302.3	1329602.8	-0.846			
N-BUTANE	Alkane	1.871	0.36	-1.47	0.000364	0.007727	C	2417753.2	6698201.3	-0.872			
DIISOBUTYL CELLOSOLVE	Ether	1.856	0.39	-1.37	0.000199	0.00485	C	1504820.3	3900547.9	-0.871	0.964	100	Detected in 100% of samples at early RT (2.72 min, solvent-front region); glycol ether chemistry consistent with background/sample-preparation contamination rather than plant-derived origin. Excluded from interpretation.
Unknown_ID158	Other	1.819	0.49	-1.04	0.000665	0.011479	C	72068.7	147702.8	-0.861			
ISOPHYTOL	C13 norisoprenoid	1.812	0.41	-1.29	0.000022	0.000897	C	179055	439165.4	-0.874			
ORTHO-TERT-BUTYLPHENOL	Aromatic	1.806	0.31	-1.67	0.001369	0.019548	C	4287.8	13623	-0.793			
6-METHYL-5-HEPTEN-2-ONE	Ketone	1.801	0.45	-1.17	0.0015	0.020363	C	249516.2	559632.9	-0.863			
Unknown_ID224	Other	1.789	0.33	-1.61	0.000662	0.011479	C	4319	13211.2	-0.791			
TERT-BUTYL 2-PICOLYL SULFIDE	Sulfur compound	1.767	0.18	-2.45	0.000006	0.000452	C	7852.3	42918.7	-0.888			
Unknown_ID317	Other	1.74	0.23	-2.14	0.0014	0.0203	C	8306.5	36508.6	-0.879			

		9			94	63							
**ionol (alpha-e-)_ID265**	**C13 norisoprenoid**	**1.731**	**0.38**	**-1.38**	**0.002761**	**0.031763**	**C**	**177634.3**	**461471**	**-0.78**			
**ionol (alpha-e-)_ID263**	**C13 norisoprenoid**	**1.731**	**0.38**	**-1.38**	**0.002762**	**0.031763**	**C**	**177634.3**	**461452.1**	**-0.78**			
**CITRONELLYL FORMATE**	**Monoterpene (incl. esters/alcohols/ketones)**	**1.709**	**0.43**	**-1.23**	**0.002012**	**0.025671**	**C**	**7300.8**	**17132.2**	**-0.842**			
**Unknown_ID402**	**Other**	**1.701**	**0.25**	**-2.02**	**0.000475**	**0.009138**	**C**	**6817.8**	**27726.3**	**-0.864**			
**dihydrosphingosine**	**Other**	**1.69**	**0.2**	**-2.3**	**0.001055**	**0.015602**	**C**	**14379.5**	**70696.6**	**-0.877**	**0.772**	**13**	**Low-confidence library match (Total score < 0.80) with low detection rate; likely co-elution misassignment. Excluded from interpretation.**
**Unknown_ID268**	**Other**	**1.656**	**0.42**	**-1.25**	**0.0014**	**0.019645**	**C**	**10720.8**	**25415.4**	**-0.76**			
**TERT-BUTYLBENZENE_ID264**	**Aromatic**	**1.6**	**0.35**	**-1.53**	**0.003758**	**0.038892**	**C**	**78386.5**	**225987.3**	**-0.676**			
**METHSUXIMIDE_ID563**	**Ketone**	**1.594**	**0.23**	**-2.1**	**0.001088**	**0.015805**	**C**	**9996**	**42744.3**	**-0.825**			
**TERT-BUTYLBENZENE_ID262**	**Aromatic**	**1.591**	**0.35**	**-1.5**	**0.004262**	**0.042257**	**C**	**79265.7**	**224537.8**	**-0.67**			
**4-(1-METHYLETHENYL)CYCLOHEXENE-1-METHANOL**	**Alcohol/other**	**1.588**	**0.38**	**-1.4**	**0.000182**	**0.004578**	**C**	**15859**	**41936.7**	**-0.819**			
**1,3,5-TRIMETHYLCYCLOHEXANE**	**Other**	**1.58**	**0.28**	**-1.85**	**0.002395**	**0.028744**	**C**	**11996.3**	**43199.7**	**-0.792**			
**ionol (alpha-e-)_ID401**	**C13 norisoprenoid**	**1.464**	**0.39**	**-1.35**	**0.000747**	**0.012122**	**C**	**16010**	**40731.8**	**-0.754**			
**eicosane_ID391**	**Alkane**	**1.405**	**0.27**	**-1.87**	**0.000959**	**0.01445**	**C**	**18108.2**	**66242.9**	**-0.737**	**One of multiple eicosane isomers/co-eluting features detected in this dataset (RT 18.84 min); only this feature met strict**		

											differential criteria. Reported as "eicosane (RT 18.84 min)" in the main text to avoid ambiguity with other eicosane-annotated features.
PROPYL FORMATE	Ester (non-terpenoid)	1.34	0.46	-1.11	0.004287	0.042257	C	8148.3	17581.7	-0.67	
LIMONENE DIOXIDE	Monoterpene (incl. esters/alcohols/ketones)	1.285	0.31	-1.69	0.004661	0.044355	C	7740.2	25042	-0.659	

Table S4-2. OPLS-DA-derived differential volatile metabolites (VIP > 1, FDR < 0.05) between Group B and Group C aroma metabolotypes of guava leaf tea.

	Compound class	VIP	Fold change (B/C)	log2FC	P value	FDR	Higher in	Mean Group B	Mean Group C	p (correlation)	Total score	• %	Fill	Note
ALPHA-PINENE_ID123	Monoterpene (incl. esters/alcohols/ketones)	1.923	72.43	6.18	0.000001	0.00009	B	110344509.7	1523498.3	0.979				
LIMONENE	Monoterpene (incl. esters/alcohols/ketones)	1.922	31.76	4.99	0	0	B	43703009.5	1375844	0.991				
BETA-PINENE_ID150	Monoterpene (incl. esters/alcohols/ketones)	1.917	16.27	4.02	0.000001	0.00009	B	2688712.3	165260.4	0.965				
Unknown_ID120	Other	1.892	61.2	5.94	0	0.000006	B	448284.5	7324.6	0.949				
2-ISOPROPYL-3-METHYLPYRAZINE	Pyrazine	1.891	14.5	3.86	0	0	B	64910.8	4478.1	0.972				
ISOBORNEOL_ID296	Monoterpene (incl.	1.889	14.54	3.86	0.000162	0.004333	B	274312.7	18871.3	0.923				

	esters/alcohols/ketones)									
**MYRTENYL ACETATE_ID277**	**Monoterpene (incl. esters/alcohols/ketones)**	**1.888**	**15.16**	**3.92**	**0**	**0**	**B**	**56115.2**	**3702.1**	**0.973**
**BETA-PINENE_ID161**	**Monoterpene (incl. esters/alcohols/ketones)**	**1.888**	**4.46**	**2.16**	**0.00038**	**0.007873**	**B**	**2815727.5**	**631044.7**	**0.887**
**(E,E)-farnesol_ID162**	**Sesquiterpene (incl. esters/alcohols)**	**1.887**	**4.16**	**2.06**	**0.000426**	**0.008398**	**B**	**1777069.5**	**427595.6**	**0.88**
**ALPHA-FENCHYL ACETATE**	**Monoterpene (incl. esters/alcohols/ketones)**	**1.876**	**7.1**	**2.83**	**0**	**0.000013**	**B**	**136412.8**	**19205.9**	**0.942**
**ALPHA-PHELLANDRENE_ID168**	**Monoterpene (incl. esters/alcohols/ketones)**	**1.875**	**5.26**	**2.4**	**0.001583**	**0.020812**	**B**	**348953**	**66312.9**	**0.788**
**ALPHA-TERPINENE_ID122**	**Monoterpene (incl. esters/alcohols/ketones)**	**1.851**	**20.97**	**4.39**	**0.0001**	**0.002956**	**B**	**247137.8**	**11783.4**	**0.933**
**Unknown_ID252**	**Other**	**1.846**	**10.13**	**3.34**	**0.000002**	**0.000135**	**B**	**38420**	**3792.4**	**0.918**
**Unknown_ID130**	**Other**	**1.838**	**14.39**	**3.85**	**0.000073**	**0.002238**	**B**	**48500.8**	**3370.3**	**0.89**
**ISOBUTYLBENZENE_ID131**	**Aromatic**	**1.832**	**5.85**	**2.55**	**0.000012**	**0.000645**	**B**	**61416.7**	**10507.3**	**0.875**
**Unknown_ID128**	**Other**	**1.831**	**9.13**	**3.19**	**0.00096**	**0.01445**	**B**	**28865.3**	**3162.9**	**0.834**
**PYRAZOLE**	**Other**	**1.827**	**6.31**	**2.66**	**0.00001**	**0.000565**	**B**	**56629**	**8974.4**	**0.881**
**CAMPHENE_ID129**	**Monoterpene (incl. esters/alcohols/ketones)**	**1.814**	**18.84**	**4.24**	**0.000041**	**0.001457**	**B**	**236732.5**	**12565.4**	**0.883**
**Unknown_ID395**	**Other**	**1.8**	**6.68**	**2.74**	**0.000319**	**0.007146**	**B**	**9007.8**	**1348.7**	**0.827**
**ISOBUTYLBENZENE_ID133**	**Aromatic**	**1.797**	**5.07**	**2.34**	**0.000035**	**0.00131**	**B**	**61416.7**	**12122.8**	**0.849**
**Unknown_ID275**	**Other**	**1.783**	**4.47**	**2.16**	**0.000016**	**0.000754**	**B**	**28791**	**6439.4**	**0.872**

**Unknown_ID73**	**Other**	**1.78**	**4.44**	**2.15**	**0.003659**	**0.038345**	**B**	**124682.5**	**28076.7**	**0.774**			
**BORNYL FORMATE**	**Monoterpene (incl. esters/alcohols/ketones)**	**1.779**	**5.08**	**2.35**	**0**	**0.000064**	**B**	**84875.5**	**16697.3**	**0.918**			
**BORNYL PROPIONATE**	**Monoterpene (incl. esters/alcohols/ketones)**	**1.774**	**4.95**	**2.31**	**0.000001**	**0.000068**	**B**	**85165.5**	**17218.1**	**0.916**			
**DIMETHYL TRISULFIDE**	**Sulfur compound**	**1.765**	**8.06**	**3.01**	**0.000534**	**0.010053**	**B**	**39848.3**	**4946.2**	**0.806**			
**3-HYDROXY-4-HYDROXYMETHYL-3,5-DIMETHYL-1,5-HEXADIENE**	**Alcohol/other**	**1.755**	**6.47**	**2.69**	**0.000013**	**0.000645**	**B**	**56491.7**	**8732.8**	**0.864**			
**neryl acetate**	**Monoterpene (incl. esters/alcohols/ketones)**	**1.755**	**4.32**	**2.11**	**0.001542**	**0.020596**	**B**	**43681.5**	**10121.6**	**0.845**			
**Y-METHYLBICYCLO (4.3.0) NON-6-EN-8-ONE**	**Other**	**1.748**	**5.92**	**2.57**	**0.000007**	**0.000452**	**B**	**16560.7**	**2797.8**	**0.869**			
**Oxybuprocaine**	**Other**	**1.734**	**3.27**	**1.71**	**0.00065**	**0.011479**	**B**	**39666**	**12140.2**	**0.836**	**0.739**	**8**	**Low-confidence library match (Total score < 0.80) with low detection rate; chemically implausible as a tea-derived compound (local anesthetic). Excluded from interpretation.**
**DIMETHYL THIOETHER**	**Sulfur compound**	**1.717**	**5.47**	**• 2.45**	**0.000336**	**0.007314**	**B**	**1696069.3**	**310106.3**	**0.829**			
**METHYL VINYL KETONE_ID12**	**Ketone**	**1.708**	**2.27**	**1.18**	**0.00005**	**0.001588**	**B**	**562877.7**	**248353.1**	**0.855**			
**BUTANE**	**Alkane**	**1.708**	**2.27**	**1.18**	**0.00005**	**0.001588**	**B**	**562877.7**	**248353.1**	**0.855**			
**Unknown_ID281**	**Other**	**1.702**	**5.19**	**2.38**	**0.000012**	**0.000645**	**B**	**16349**	**3151.2**	**0.873**			
**Unknown_ID305**	**Other**	**1.698**	**3.24**	**1.7**	**0.000026**	**0.001038**	**B**	**15380.5**	**4739.9**	**0.837**			

**Unknown_ID49**	**Other**	**1.677**	**4.02**	**2.01**	**0.003074**	**0.033932**	**B**	**6466**	**1607.9**	**0.75**	
**Unknown_ID278**	**Other**	**1.676**	**4.78**	**2.26**	**0.000021**	**0.000897**	**B**	**40790.2**	**8528.3**	**0.851**	
**Unknown_ID292**	**Other**	**1.631**	**4.64**	**2.21**	**0.000859**	**0.013681**	**B**	**17221**	**3709.2**	**0.766**	
**Unknown_ID135**	**Other**	**1.625**	**5.5**	**2.46**	**0.000683**	**0.011544**	**B**	**15027.3**	**2729.9**	**0.843**	
**Unknown_ID374**	**Other**	**1.622**	**3.24**	**1.7**	**0.002217**	**0.0272**	**B**	**10265.2**	**3166.7**	**0.796**	
**Unknown_ID164**	**Other**	**1.616**	**2.62**	**1.39**	**0.004413**	**0.042961**	**B**	**56907.7**	**21750.2**	**0.72**	
**(E)-3,7-DIMETHYL-2,6-OCTADIEN-1-OL**	**Other**	**1.615**	**3.2**	**1.68**	**0.000123**	**0.00351**	**B**	**72040.8**	**22545.7**	**0.801**	
**Unknown_ID279**	**Other**	**1.61**	**5.13**	**2.36**	**0.000042**	**0.001457**	**B**	**16349.3**	**3188.9**	**0.834**	
**hexacosane_ID473**	**Alkane**	**1.598**	**3.12**	**1.64**	**0.000171**	**0.004432**	**B**	**72091.5**	**23096.2**	**0.791**	
**(1S-TRANS)-2-METHYL-5-(1-METHYLETHENYL)-2-CYCLOHEXEN-1-OL_ID443**	**Other**	**1.592**	**2.65**	**1.41**	**0.00071**	**0.011757**	**B**	**15318.2**	**5781.1**	**0.775**	
**Unknown_ID69**	**Other**	**1.549**	**4.87**	**2.28**	**0.002234**	**0.0272**	**B**	**54182.5**	**11129.8**	**0.822**	
**Unknown_ID125**	**Other**	**1.542**	**5.48**	**2.45**	**0.00285**	**0.032327**	**B**	**33760.3**	**6161.8**	**0.672**	
**(R)-2-METHYL-5-(1-METHYLETHENYL)-2-CYCLOHEXEN-1-ONE**	**Ketone**	**1.507**	**3.41**	**1.77**	**0.000638**	**0.011479**	**B**	**17692.3**	**5193.1**	**0.765**	
**Unknown_ID124**	**Other**	**1.457**	**6.48**	**2.7**	**0.003528**	**0.037456**	**B**	**55200.5**	**8517.1**	**0.733**	
**sesquisabinene**	**Sesquiterpene (incl. esters/alcohols)**	**1.889**	**0.2**	**-2.33**	**0.003354**	**0.036071**	**C**	**2301128.5**	**11580527.7**	**-0.823**	**Same peak as CIS-NEROLIDYL ACETATE (RT diff 0.002 min); count once**
**CIS-NEROLIDYL ACETATE**	**Sesquiterpene (incl. esters/alcohols)**	**1.889**	**0.2**	**-2.33**	**0.003354**	**0.036071**	**C**	**2301128.5**	**11580527.7**	**-0.823**	**Same peak as sesquisabinene (RT diff 0.002 min); count once**

Unknown_ID267	Other	1.884	0.41	-1.29	0.002148	0.026952	C	301066	736918.4	-0.866			
BETA-BUTYROLACTONE	Lactone	1.881	0.41	-1.27	0.000406	0.008194	C	255765.2	617465.7	-0.853			
METHYL 4-METHYL-3-CYCLOHEXENYL KETONE	Ketone	1.88	0.35	-1.53	0.004462	0.042961	C	459302.3	1329602.8	-0.846			
N-BUTANE	Alkane	1.871	0.36	-1.47	0.000364	0.007727	C	2417753.2	6698201.3	-0.872			
DIISOBUTYL CELLOSOLVE	Ether	1.856	0.39	-1.37	0.000199	0.00485	C	1504820.3	3900547.9	-0.871	0.964	100	Detected in 100% of samples at early RT (2.72 min, solvent-front region); glycol ether chemistry consistent with background/sample-preparation contamination rather than plant-derived origin. Excluded from interpretation.
Unknown_ID158	Other	1.819	0.49	-1.04	0.000665	0.011479	C	72068.7	147702.8	-0.861			
ISOPHYTOL	C13 norisoprenoid	1.812	0.41	-1.29	0.000022	0.000897	C	179055	439165.4	-0.874			
ORTHO-TERT-BUTYLPHENOL	Aromatic	1.806	0.31	-1.67	0.001369	0.019548	C	4287.8	13623	-0.793			
6-METHYL-5-HEPTEN-2-ONE	Ketone	1.801	0.45	-1.17	0.0015	0.020363	C	249516.2	559632.9	-0.863			
Unknown_ID224	Other	1.789	0.33	-1.61	0.000662	0.011479	C	4319	13211.2	-0.791			
TERT-BUTYL 2-PICOLYL SULFIDE	Sulfur compound	1.767	0.18	-2.45	0.000006	0.000452	C	7852.3	42918.7	-0.888			
Unknown_ID317	Other	1.749	0.23	-2.14	0.001494	0.020363	C	8306.5	36508.6	-0.879			
ionol (alpha-e-)_ID265	C13 norisoprenoid	1.731	0.38	-1.38	0.002761	0.031763	C	177634.3	461471	-0.78			
ionol (alpha-e-)_ID263	C13 norisoprenoid	1.731	0.38	-1.38	0.002762	0.031763	C	177634.3	461452.1	-0.78			
CITRONELLYL FORMATE	Monoterpene (incl.	1.709	0.43	-1.23	0.002012	0.025671	C	7300.8	17132.2	-0.842			

	**esters/alcohols/ketones)**												
**Unknown_ID402**	**Other**	**1.701**	**0.25**	**-2.02**	**0.000475**	**0.009138**	**C**	**6817.8**	**27726.3**	**-0.864**			
**dihydrosphingosine**	**Other**	**1.69**	**0.2**	**-2.3**	**0.001055**	**0.015602**	**C**	**14379.5**	**70696.6**	**-0.877**	**0.772**	**13**	**Low-confidence library match (Total score < 0.80) with low detection rate; likely co-elution misassignment. Excluded from interpretation.**
**Unknown_ID268**	**Other**	**1.656**	**0.42**	**-1.25**	**0.0014**	**0.019645**	**C**	**10720.8**	**25415.4**	**-0.76**			
**TERT-BUTYLBENZENE_ID264**	**Aromatic**	**1.6**	**0.35**	**-1.53**	**0.003758**	**0.038892**	**C**	**78386.5**	**225987.3**	**-0.676**			
**METHSUXIMIDE_ID563**	**Ketone**	**1.594**	**0.23**	**-2.1**	**0.001088**	**0.015805**	**C**	**9996**	**42744.3**	**-0.825**			
**TERT-BUTYLBENZENE_ID262**	**Aromatic**	**1.591**	**0.35**	**-1.5**	**0.004262**	**0.042257**	**C**	**79265.7**	**224537.8**	**-0.67**			
**4-(1-METHYLETHENYL)CYCLOHEXENE-1-METHANOL**	**Alcohol/other**	**1.588**	**0.38**	**-1.4**	**0.000182**	**0.004578**	**C**	**15859**	**41936.7**	**-0.819**			
**1,3,5-TRIMETHYLCYCLOHEXANE**	**Other**	**1.58**	**0.28**	**-1.85**	**0.002395**	**0.028744**	**C**	**11996.3**	**43199.7**	**-0.792**			
**ionol (alpha-e-)_ID401**	**C13 norisoprenoid**	**1.464**	**0.39**	**-1.35**	**0.000747**	**0.012122**	**C**	**16010**	**40731.8**	**-0.754**			
**eicosane_ID391**	**Alkane**	**1.405**	**0.27**	**-1.87**	**0.000959**	**0.01445**	**C**	**18108.2**	**66242.9**	**-0.737**	**One of multiple eicosane isomers/co-eluting features detected in this dataset (RT 18.84 min); only this feature met strict differential criteria. Reported as**		

											**"eicosane (RT 18.84 min)" in the main text to avoid ambiguity with other eicosane-annotated features.**
**PROPYL FORMATE**	**Ester (non-terpenoid)**	**1.34**	**0.46**	**-1.11**	**0.004287**	**0.042257**	**C**	**8148.3**	**17581.7**	**-0.67**	
**LIMONENE DIOXIDE**	**Monoterpene (incl. esters/alcohols/ketones)**	**1.285**	**0.31**	**-1.69**	**0.004661**	**0.044355**	**C**	**7740.2**	**25042**	**-0.659**	

Note: Discriminant volatiles were prioritized using VIP > 1, $|log_2FC| \geq 1$, $p < 0.05$ and FDR < 0.05, with p values from Welch's t-tests on $log_2(x + 1)$-transformed data. Because these tests treat biological replicates as observations whereas group membership is defined at the cultivar level, p and FDR values serve as screening criteria for ranking features rather than as cultivar-level inference of group differences (see Table S1). Group B = 'Yanzhi Hong', 'Jindou Xiang'; Group C = 'Xigua No. 2', 'Hongbaoshi', 'Xigua'.

Table S5. Monoterpene and total volatile abundance of the eight cultivars.

Cultivar	GC–MS aroma group	16 monoterpene markers (×10^{8})	Total volatiles (×10^{9})	Monoterpene markers (% of total)
Yanzhi Hong	B	5.06 ± 0.64	2.60 ± 0.22	19.5
Jindou Xiang	B	2.62 ± 0.38	1.59 ± 0.14	16.5
Xinshiji	A	2.74 ± 0.11	1.85 ± 0.08	14.8
Fenhongmi	A	1.63 ± 0.17	1.56 ± 0.11	10.5
Wuzi	A	1.40 ± 0.21	1.70 ± 0.25	8.2
Xigua	C	1.90 ± 0.18	1.85 ± 0.20	10.2
Xigua No. 2	C	1.95 ± 0.25	1.95 ± 0.19	10.0
Hongbaoshi	C	1.38 ± 0.14	1.50 ± 0.14	9.2

Note: Values are sums of raw HS-GC–MS peak areas (mean ± SD, n = 3 biological replicates). Monoterpene markers are the 16 monoterpene-related markers used for the monoterpene pathway score (Figure 8); their percentage is expressed relative to total volatile peak area.

Table S6. Structural-confidence summary of metabolites underlying the pathway/product-class scores used in the integrated analysis.

Pathway/product-class score	Total markers (n)	Level 1: confirmed by authentic standards (n)	Level 2: putatively annotated (n)	Representative Level 1 compounds
Monoterpene volatiles	16	6	10	α-Pinene; β-Pinene; Limonene; γ-Terpinene; Linalool; α-Terpineol
Sesquiterpene volatiles	10	0	10	None
Ketone/lactone/alkane/aromatic volatiles	10	0	10	None
Carotenoid-derived volatiles	4	0	4	None
Flavonoid glycosides	30	3	27	Isoquercitrin; Guajavarin; Avicularin
Ellagitannins / hydrolysable tannins	4	0	4	None
Triterpenoid acids / saponin-related candidates	5	1	4	Euscaphic acid
Flavonol antioxidant candidates	4	1	3	Astragalin

Note: Level 1 indicates confirmation with an authentic reference standard under the same analytical conditions. Level 2 indicates putative annotation without authentic-standard confirmation. For LC–MS features, Level 2 annotations were based on accurate mass and diagnostic MS/MS matching; GC–MS volatile annotations were based on NIST spectral matching together with retention-index agreement and are treated here as putative rather than authentic-standard-confirmed.

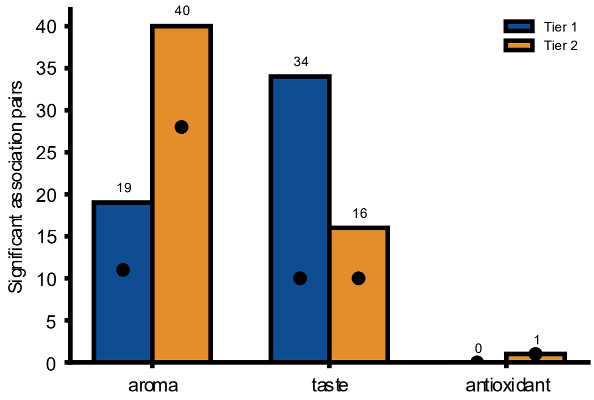

Figure S1. Candidate reliability-tier distribution across quality domains. Bar heights show significant association pairs assigned to Tier 1 or Tier 2 within aroma, taste, and antioxidant-related analyses. Black dots indicate the corresponding number of unique metabolites. This figure supports the integrated candidate prioritization summarized in Figure 7.

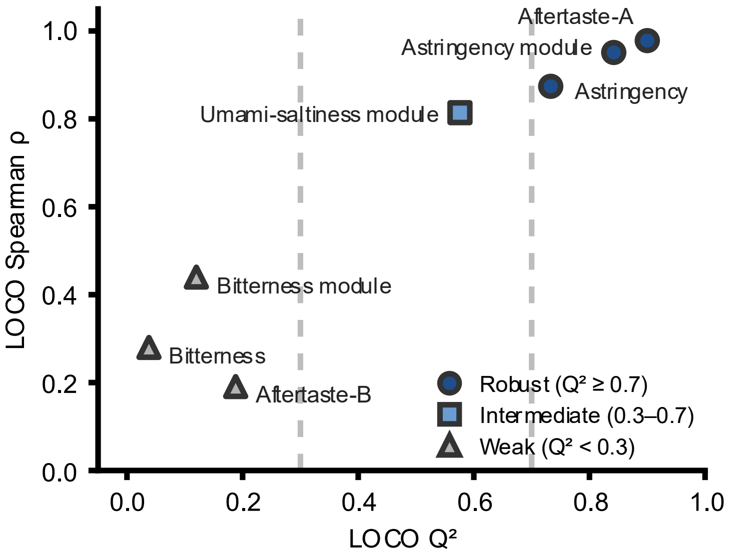

Figure S2. Taste sPLS model performance under leave-one-cultivar-out (LOCO) validation. Points show LOCO Q^2 and LOCO Spearman ρ for electronic-tongue traits and taste modules. Colours denote model performance classes defined by LOCO Q^2 (robust, $Q^2 \geq 0.7$; intermediate, $0.3 \leq Q^2 < 0.7$; weak, $Q^2 < 0.3$). Cultivar-level permutation p values (999 permutations with the three replicates of each cultivar permuted as a block) are reported in the main text. The figure supports the Figure 7b evidence strip and the main-text emphasis on astringency-related models.

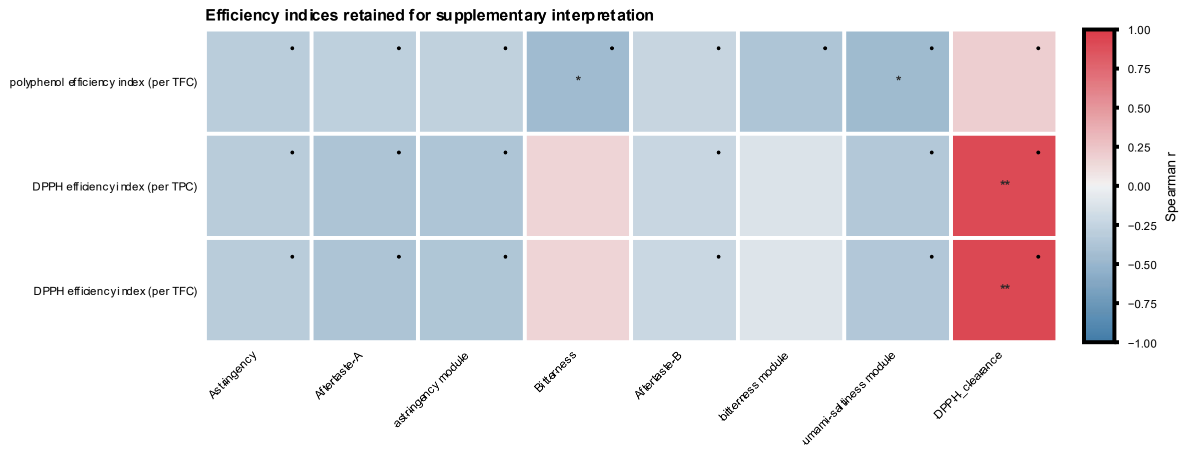

Figure S3. Efficiency-index heatmap retained for supplementary interpretation.

The displayed variables are derived efficiency indices, including DPPH efficiency index (per TPC), DPPH efficiency index (per TFC), and polyphenol efficiency index (per TFC). Cell color indicates Spearman correlation coefficients (r), asterisks indicate p/FDR support, and black marks indicate LOCO-robust associations. These indices are derived quantities and are not interpreted as independent predictors.

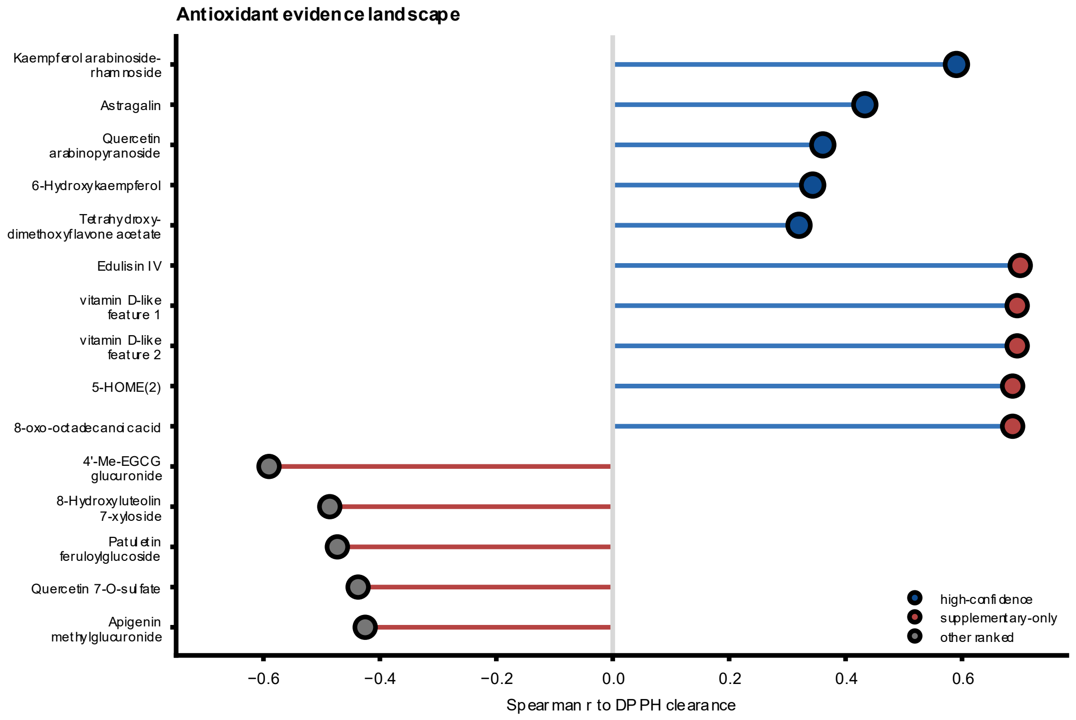

Figure S4. Antioxidant evidence landscape for DPPH-centered candidate prioritization. Candidate metabolites are ranked by integrated evidence score and positioned by Spearman

correlation with DPPH clearance. Point size reflects evidence score, and supplementary-only variables are distinguished from high-confidence main-text candidates. UHPLC-qTOF-MS candidates without standard confirmation should be interpreted as putatively annotated. Associations should not be interpreted as direct causality.

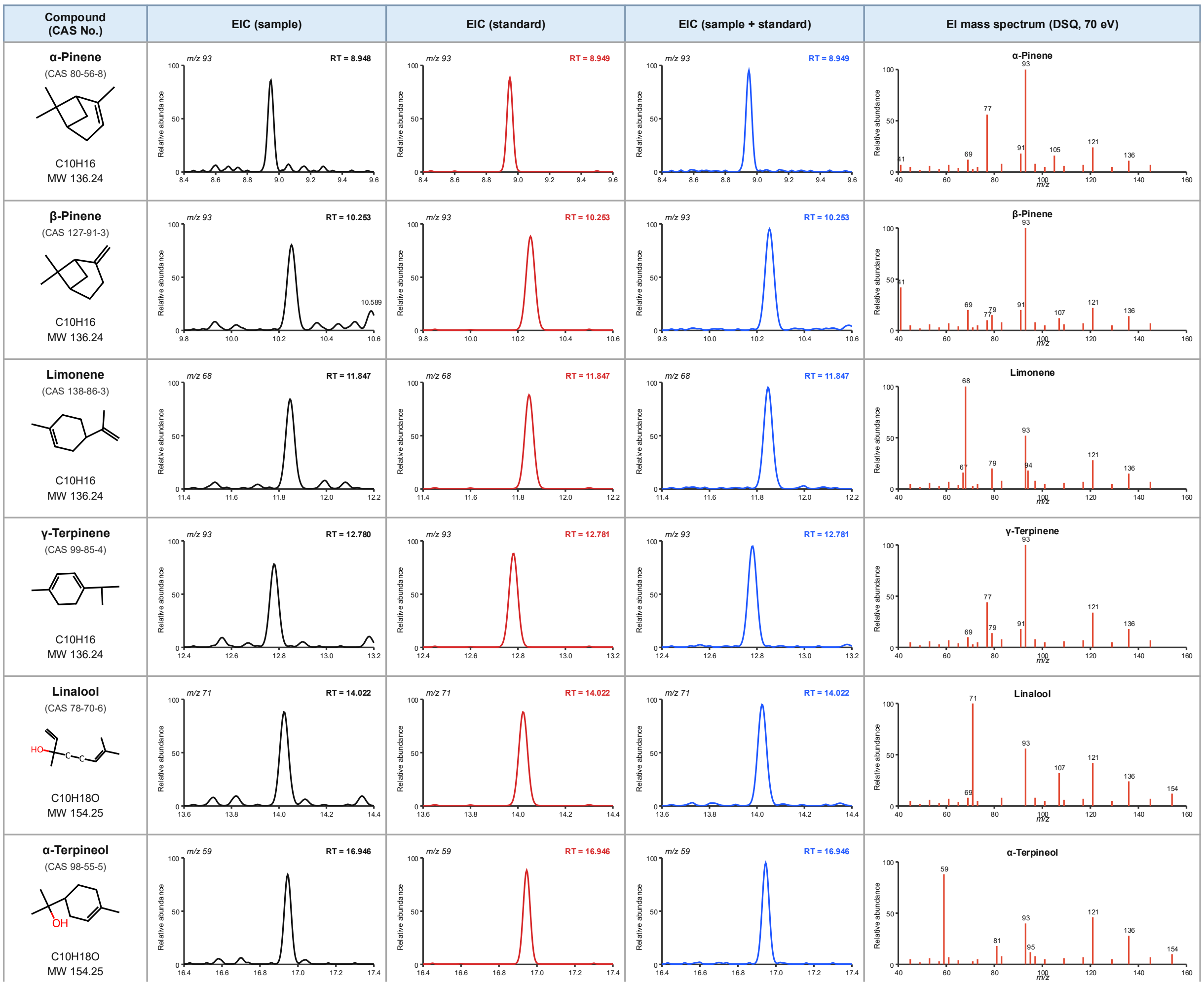

Figure S5-1 Authentic-standard confirmation of representative monoterpenes and oxygenated monoterpenes detected by GC–MS. Extracted-ion chromatograms (EICs) of the sample, authentic standard, and spiked sample are shown together with the corresponding EI mass spectra acquired on the Thermo DSQ GC–MS system under 70 eV electron ionization. The six representative compounds were α-pinene, β-pinene, limonene, γ-terpinene, linalool, and α-terpineol.

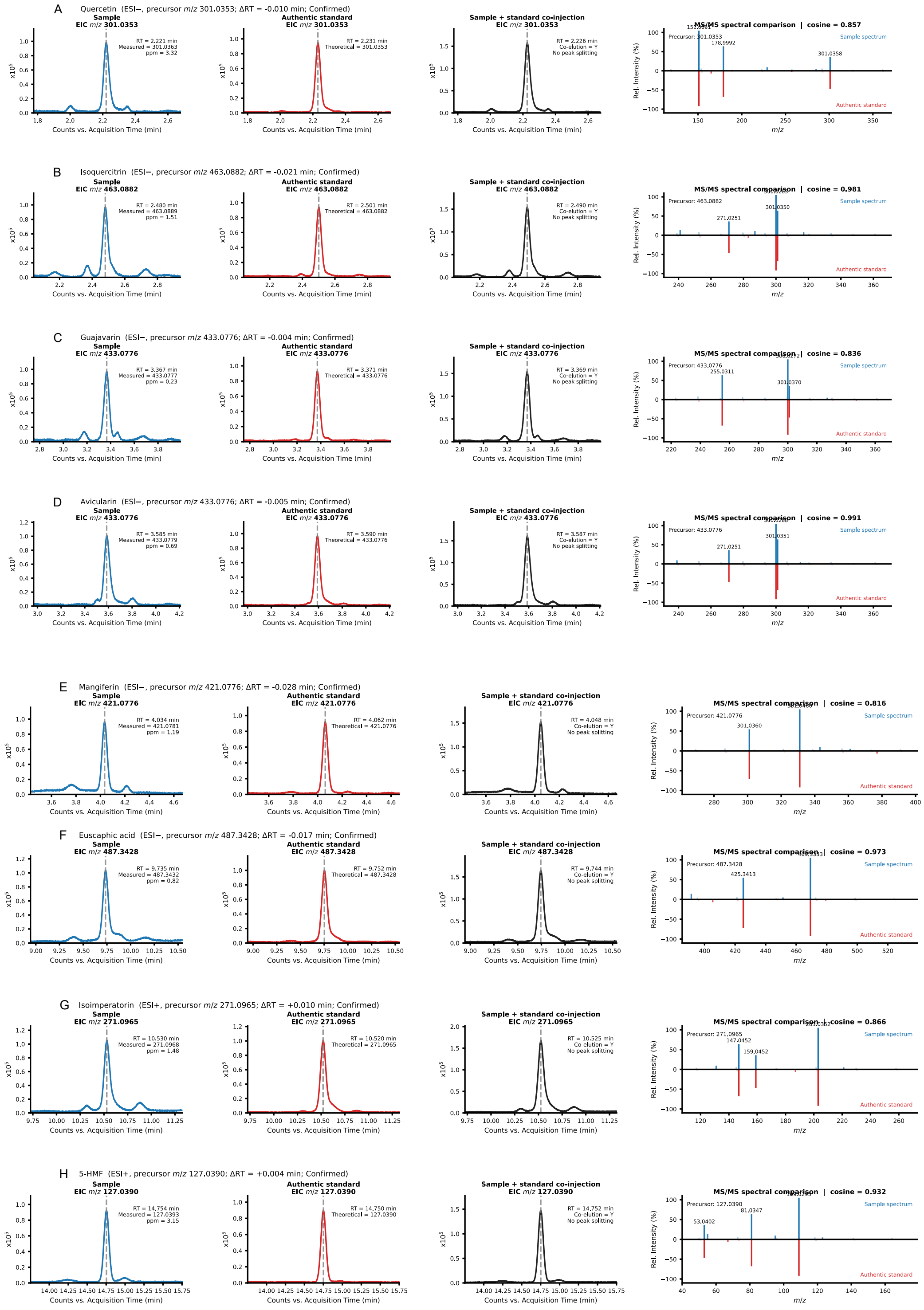

Figure S 5-2 Confirmation of selected metabolites by authentic reference standards using LC–QTOF–MS. (A–E) Catechin, quercetin, isoquercitrin, guajavarin, and avicularin. (F–J) Mangiferin, robinetin, euscaphic acid, isoimperatorin, and 5-hydroxymethylfurfural (5-HMF). For each metabolite, extracted ion chromatograms (EICs) obtained from the sample, authentic reference standard, and sample–standard co-injection are presented together with the corresponding MS/MS

spectral comparison. Note: EICs were extracted using the theoretical precursor ion of each compound under the corresponding positive- or negative-ion electrospray mode. Co-injection was considered supportive of identity when standard addition increased the target peak without producing a distinguishable secondary peak or peak splitting at the corresponding retention time.

Supplementary Methods. Compound class assignment and Shannon effective category calculation

Shannon effective category ($H' = -\Sigma p_i \ln p_i$) was calculated using class-level relative abundance proportions (p_i) of annotated compound classes, where each annotated metabolite was assigned to one of 28 compound classes (e.g., monoterpenes, sesquiterpene esters, alkanes, ketones). Abundance-weighted category counts (exp H') reflect both the number of compound classes present and the evenness of their abundance distribution. Sub-classes (e.g., monoterpene hydrocarbons vs. oxygenated monoterpenes) were aggregated at the major class level; no further subclass splitting was applied. The calculation used individual metabolite peak areas summed by class, not binary presence/absence.
